# The Dual Role of Oxidative Stress in Atherosclerosis and Coronary Artery Disease: Pathological Mechanisms and Diagnostic Potential

**DOI:** 10.3390/antiox14030275

**Published:** 2025-02-26

**Authors:** Marcin Myszko, Jerzy Bychowski, Elżbieta Skrzydlewska, Wojciech Łuczaj

**Affiliations:** 1 Department of Cardiology, Bialystok Regional Hospital, M. Skłodowskiej-Curie 25, 15-950 Bialystok, Poland; marcinmyszko@interia.pl (M.M.); kardiologia@sniadecja.pl (J.B.); 2 Department of Analytical Chemistry, Medical University of Bialystok, Mickiewicza 2d, 15-222 Bialystok, Poland; elzbieta.skrzydlewska@umb.edu.pl

**Keywords:** acute coronary syndrome, antioxidants, atherosclerosis, oxidative stress, reactive oxygen species

## Abstract

Oxidative stress plays a pivotal role in the pathogenesis of atherosclerosis and coronary artery disease (CAD), with both beneficial and detrimental effects on cardiovascular health. On one hand, the excessive production of reactive oxygen species (ROS) contributes to endothelial dysfunction, inflammation, and vascular remodeling, which are central to the development and progression of CAD. These pathological effects drive key processes such as atherosclerosis, plaque formation, and thrombosis. On the other hand, moderate levels of oxidative stress can have beneficial effects on cardiovascular health. These include regulating vascular tone by promoting blood vessel dilation, supporting endothelial function through nitric oxide production, and enhancing the immune response to prevent infections. Additionally, oxidative stress can stimulate cellular adaptation to stress, promote cell survival, and encourage angiogenesis, which helps form new blood vessels to improve blood flow. Oxidative stress also holds promise as a source of biomarkers that could aid in the diagnosis, prognosis, and monitoring of CAD. Specific oxidative markers, such as malondialdehyde (MDA), isoprostanes (isoP), ischemia-modified albumin, and antioxidant enzyme activity, have been identified as potential indicators of disease severity and therapeutic response. This review explores the dual nature of oxidative stress in atherosclerosis and CAD, examining its mechanisms in disease pathogenesis as well as its emerging role in clinical diagnostics and targeted therapies. The future directions for research aimed at harnessing the diagnostic and therapeutic potential of oxidative stress biomarkers are also discussed. Understanding the balance between the detrimental and beneficial effects of oxidative stress could lead to innovative approaches in the prevention and management of CAD.

## 1. Atherosclerosis, Its Epidemiology and Related Diseases

**Figure 1:**
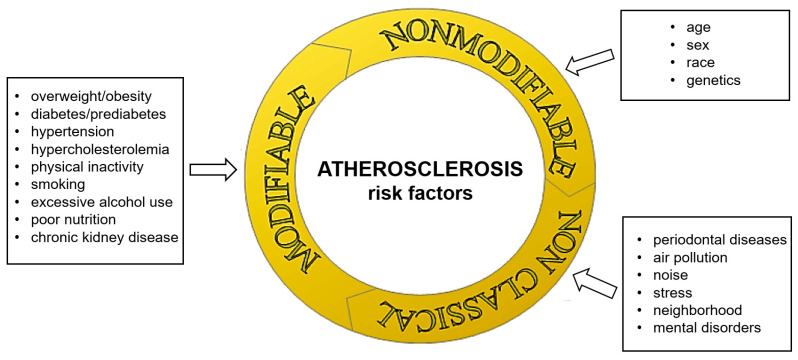
Risk factors for atherosclerosis.

Atherosclerosis is a chronic inflammatory process affecting large and medium-sized arteries. It involves the deposition of lipids in the intima of arteries, the accumulation of both physiological and modified immune system cells and smooth muscle cells, as well as the accumulation of cell breakdown products, which leads to the formation of the atherosclerotic plaques. As a consequence, the biological properties of the vessel are lost and its lumen is gradually reduced, which results in insufficient supply of tissues with oxygen and nutrients [1]. The development of diseases resulting from atherosclerosis depends on many factors. The most important, known to date risk factors for the development of atherosclerosis are shown in Figure 1, including “poor nutrition”, which is understood as both caloric imbalance (either excess or deficiency) and insufficient intake of essential micronutrients, such as vitamins and minerals.

The literature data indicate that the atherosclerotic process starts in the fetal period as shown in a postmortem study of 82 aortas taken from aborted fetuses and premature infants (mean fetal age 6.2 ± 1.3 months) who died up to 12 h after birth. Atherosclerosis affects vessels in different locations in the human body, and, consequently, their diverse clinical manifestation (Table 1).

Atherosclerosis remains one of the leading causes of death worldwide [2]. Coronary artery disease resulting from atherosclerosis is the most common single cause of death in the world, and stroke, which is also most often caused by atherosclerosis, is in second place in this inglorious ranking. These two diseases alone are responsible for over 27% of deaths worldwide. However, it is estimated that diseases resulting from atherosclerosis may be responsible for approximately 50% of deaths in developed countries [3].

**Figure 2:**
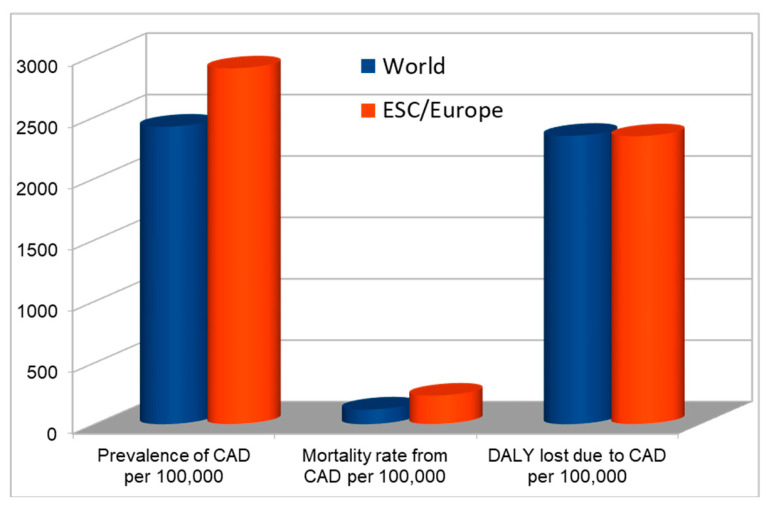
Prevalence, mortality, and DALY rates for CAD in 2019 [2,5].

Epidemiological data from 2019, the year of the beginning of the SARS-CoV-2 pandemic, indicate that CAD, on a global scale, affected approximately 200 million people [4], and was the most common cause of death, causing the death of 5.0 million men and 4.2 million women (approx. 109/100 thousand), ahead of stroke and COPD [4], with a DALY (disability-adjusted life years) rate of 2346/100 thousand. (Figure 2) [2].

According to data from the ESC (European Society of Cardiology), in the countries associated with this organization, CAD is responsible for 45.8 million DALYs (2342/100,000), and this rate is twice as high in men than in women (3262 vs. 1616/100,000) [5].

### CAD—Definition, Clinical Manifestations, and Challenges in Diagnosis and Treatment

**Figure 3:**
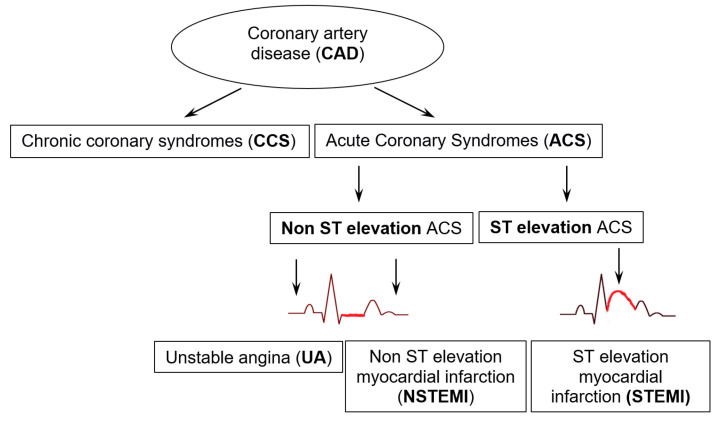
Clinical presentations of CAD. The ST segment is marked in red on the ECG diagrams.

In this article, when referring to CAD, it is understood to include both asymptomatic patients with confirmed coronary atherosclerosis, as well as chronic and acute diseases associated with coronary atherosclerosis and its consequences. A wide spectrum of diseases associated with CAD, from its suspicion based on symptoms or left ventricular dysfunction, through patients after revascularization, specific forms of CAD such as vasospastic or microvascular angina, as well as asymptomatic disease detected during screening, are classified as chronic coronary syndromes (CCSs) (Figure 3). This term was introduced by the ESC in 2019 [6].

Acute coronary syndromes (ACSs) refer to a group of conditions resulting from a sudden or progressively increasing restriction or cessation of blood flow in the coronary arteries. When there is no myocardial cell necrosis due to the disturbance in blood flow, unstable angina (UA) is diagnosed. However, if recent damage to the heart muscle is indicated by an elevated level of troponin in the blood—a protein part of the contractile apparatus—a myocardial infarction (MI) is diagnosed. To confirm a myocardial infarction, the troponin level must be accompanied by at least one clinical criterion [7]. The distinction between ST-elevation myocardial infarction (STEMI) and non-ST-elevation myocardial infarction (NSTEMI) is made based on ECG findings (Figure 3).

Validated diagnostic and treatment protocols are available for various forms of atherosclerosis and its complications. However, specialized tests such as Doppler ultrasonography, angioCT, and coronary angiography are costly, time-consuming, and require skilled personnel. Modern biomarkers, like troponin, have significantly advanced diagnostics, but clinical practice has also revealed their limitations. For instance, while troponin demonstrates nearly 100% sensitivity in diagnosing recent cardiomyocyte damage, it lacks sufficient specificity and cannot distinguish the underlying cause of the damage [8,9]. Although reperfusion treatment is well-established, therapies aimed at protecting cardiomyocytes remain at an experimental level, both as a preliminary treatment before reperfusion and in terms of preventing reperfusion injury [10].

Due to the above, new, cheap, and widely available markers are still being sought that would allow for the non-invasive monitoring of the progression of the disease and better predict the risk of complications in the course of acute atherosclerosis-related diseases, especially CAD. Among the biochemical parameters, monitoring and therapy targets have been established for the assessment of LDL cholesterol fraction (ESC class I recommendations), while recent studies continue to provide relevant information on the importance of other lipid compounds and their metabolites, which may translate into an earlier diagnosis of atherosclerosis-related diseases and a better assessment of the effectiveness of patient treatment. Therefore, further research is being conducted aimed at important elements of the pathogenesis of CAD, including changes in the lipidome dependent on oxidative stress and inflammation [8]. In this review, in addition to presenting an outline of the pathogenesis and sources of ROS involved in the development of atherosclerosis, the potential usefulness of lipid, protein, and nucleic acid peroxidation products as biomarkers in clinical practice is also highlighted.

## 2. Pathophysiology of Atherosclerosis and CAD

**Figure 4:**
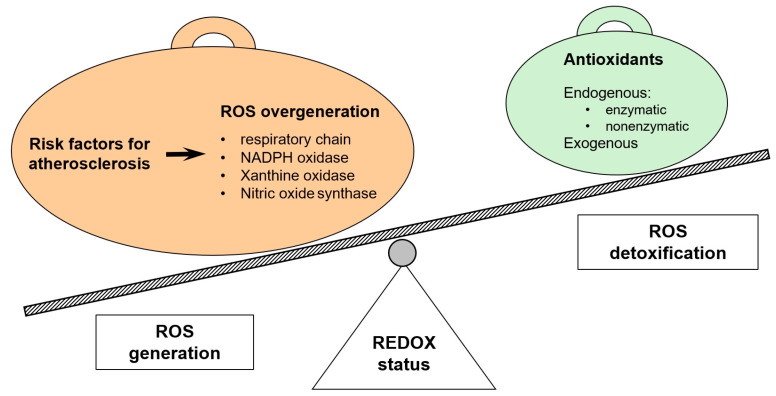
The concept of oxidative stress as a disorder underlying the development of atherosclerosis.

A permanent physiological element, playing a regulatory role in the human body, are ROS, which include the superoxide anion radical (O_2_^•−^), hydrogen peroxide (H_2_O_2_), hydroxyl radical (OH^•^), and peroxynitrite (ONOO^−^). ROS have an unpaired electron in their valence shell, which determines their high reactivity. ROS are physiologically formed in low concentrations and have a short half-life, which makes them perfect as secondary messengers [11], and their production and action is inhibited by components of the antioxidant system. Antioxidants can be divided into an endogenous type, which includes enzymatic and non-enzymatic varieties, and an exogenous type. The main enzymatic antioxidants include superoxide dismutase (SOD), catalase (CAT), glutathione peroxidase (GPx), ceruloplasmin, and thioredoxin. Ceruloplasmin acts as an antioxidant by limiting free iron, reducing iron-driven oxidative damage. In the heart, it protects cells from oxidative injury, especially during inflammation or ischemia. Altered ceruloplasmin levels have been linked to cardiovascular disease, suggesting its role as both a marker and modulator of oxidative stress in heart tissues [12]. Endogenous non-enzymatic antioxidants are metal binding proteins, including glutathione (GSH), uric acid (UA), melatonin, bilirubin, albumin, and polyamines. On the other hand, antioxidants that are supplied with food, called exogenous, include vitamins A, C, E, and K, carotenoids, polyphenols, and elements such as selenium, iron, zinc, and magnesium [13]. When the amount of ROS produced exceeds the capacity of their inactivation by the components of the antioxidant system, oxidative stress occurs, characterized by a shift in the body’s redox balance towards oxidation processes. This situation is closely related to the development of atherosclerosis [11], which is schematically presented in Figure 4. It has been found that already at the stage of the appearance of atherosclerosis risk factors, there is an overproduction of ROS and a decrease in the antioxidant capacity of the body, which promotes the occurrence of oxidative stress.

Risk factors for the development of atherosclerosis can therefore be the cause of oxidative stress. In arterial hypertension, through various mechanisms, including the action of angiotensin II (ATII), aldosterone, endothelin I (ETI), and the increased tone of the sympathetic system, oxidases are activated, in particular NADPH oxidase, which results in the increased generation of ROS in the vascular system [14]. In addition, carbohydrate metabolism disorders, especially diabetes, lead to an increase in ROS production [15]. In obesity, excessive adipose tissue, which is hormonally and humourally active, contributes to the increased production of adipokines and pro-inflammatory cytokines such as leptin, resistin, IL-6, and TNF-α, causing chronic inflammation and increased generation of ROS [16]. In addition, the cytokine-activated transcription factor NF-κB directly stimulates the production of ROS [17]. Cigarette smoke is both a direct source of ROS and an activator of endogenous mechanisms of their generation, which is visible in the population of patients hospitalized due to coronary heart disease, in whom a relationship between smoking and the presence of oxidative stress markers was demonstrated [18]. In addition, hypercholesterolemia is associated with increased levels of oxidative stress markers with simultaneous impairment of antioxidant mechanisms [19]. Also, low levels of physical activity are associated with increased levels of markers of oxidative stress. The introduction of standardized exercise programs leads to a reduction in the level of these markers and an increase in the level/activity of the parameters of the endogenous antioxidant system [20]. It should be emphasized that increased ROS synthesis in muscle tissue is a physiological response to physical exercise [21].

The above-mentioned factors promoting the development of atherosclerosis consequently lead to the activation of vascular endothelial cells, as a result of which it loses its beneficial physiological properties (Table 2) and promotes local inflammation.

Endothelial activation leads to the adhesion and activation of immune system cells, in particular monocytes and T lymphocytes. The loss of tightness of the endothelial barrier promotes the passage of monocytes and the penetration of oxidized low-density lipoproteins (oxLDLs) from the lumen of the vessel to its wall. Monocytes, leaving the lumen of the vessel, transform into macrophages and absorb oxLDLs, becoming the foam cells. At the same time, activated immune cells, platelets, and endothelium, produce pro-inflammatory cytokines, chemokines, growth factors, and ROS. Foam cells die over time, forming the lipid core of the plaque [22]. Vascular smooth muscle cells (VSMCs) activated under the influence of the chemokines and adhesion factors are responsible for the formation of the fibrous part, including the stabilizing cap. These cells, moving to the inflammatory site, produce, among others, collagen and elastin, which stabilize the developing atherosclerotic plaque [23].

**Figure 5:**
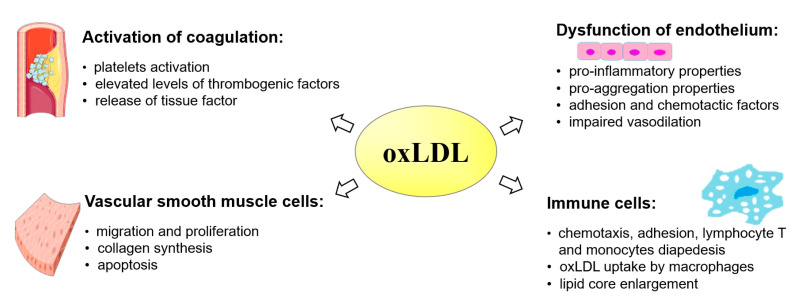
Involvement of oxLDLs in the pathogenesis of atherosclerosis and activation of metalloproteinases, (MMPs).

OxLDLs participate in numerous metabolic processes leading to the development of atherosclerosis (Figure 5), including enhancing the expression of adhesion molecules such as ICAM-1 (intracellular adhesion molecule-1) or VCAM-1 (vascular cell adhesion molecule-1) for monocytes on the endothelial surface, and chemotaxis by increasing the expression of MCP1 (macrophage chemotactic protein 1) [24]. OxLDLs themselves are also a growth factor for macrophages [25]. They enhance the migration and proliferation of vascular smooth muscle cells (VSMCs) by intensifying the production of platelet-derived growth factor (PDGF) and basic fibroblast growth factor (FGF) in the endothelium and macrophages, as well as collagen synthesis in VSMCs [26]. In addition to these effects, oxLDLs activate metalloproteinases (MMPs) that degrade the fibrous cap of atherosclerotic plaque [27], enhance platelet aggregation, and reduce plasma fibrinolytic activity [28].

Under the influence of growth factors such as vascular endothelial growth factor (VEGF), blood vessels proliferate into the plaque, which increase the volume and lead to destabilization of the plaque through the influx of cells of the immune system, activation of inflammation, and, due to the fragility of the vessels, through hemorrhages into the plaque [29]. Additionally, the intensification of inflammation inhibits the production of fibrous elements of the extracellular matrix and, through the activity of metalloproteinases, causes the degradation of collagen and elastin. Under the influence of inflammatory mediators, smooth muscle cells transform into osteoblast-like or osteocyte-like cells and calcium begins to be deposited in the atherosclerotic plaque. Active inflammation, through mediators (e.g., TNFα, IL-6), causes the accumulation of material in the core of the plaque in the process called microcalcification, which is considered a feature of its instability. The suppression of inflammation leads to a process called macrocalcification, which leads to the stabilization of the plaque [30].

Damage to the atherosclerotic plaque, in the form of a rupture or erosion, is the most common cause of acute complications of atherosclerosis, which include, among others, ACS. Oxidative stress, which causes lipid peroxidation and maintains inflammation, is the basis of processes leading to the formation and destabilization of atherosclerotic plaque [31]. Several ROS-related factors act as triggers for plaque instability and rupture (Table 3).

**Figure 6:**
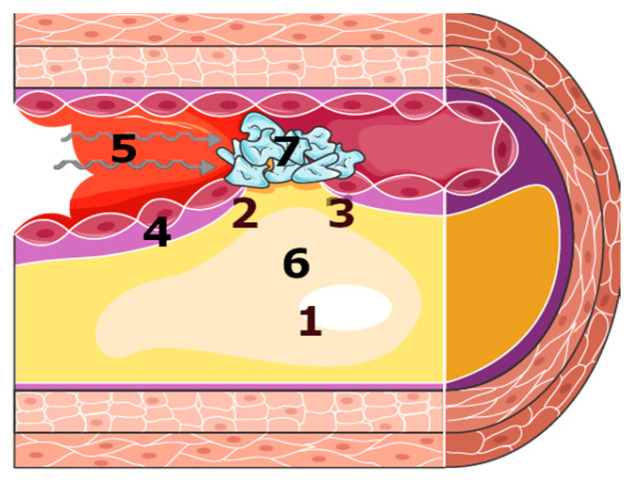
Multifactorial pathogenesis of acute coronary syndrome. 1. Large lipid core. 2. Fracture of the plaque cover. 3. Metalloproteinases degrade the connective tissue cover of fibrous cap. 4. Dysfunctional endothelium. 5. Mechanical forces acting on the plaque. 6. Thrombogenic plaque content. 7. Platelet activation—clot on a ruptured plaque.

The endothelium, activated by the inflammatory process, ROS, and oxLDLs, loses its protective properties, making it easier for physical factors (vessel wall stress, shear stress) to damage the plaque (Figure 6). With a deficiency of anticoagulant factors (which the activated endothelium cannot provide), a thrombus forms on the damaged plaque and suddenly reduces or stops the flow in the artery [22]. When the oxygen supply in the blood is insufficient to meet the metabolic demands of cardiomyocytes (due to restricted blood flow) or when oxygen delivery is completely blocked (due to inhibited blood flow), the contractile function of the cardiomyocytes is impaired. If this condition persists, cardiomyocyte necrosis will occur, the clinical manifestation of which is myocardial infarction.

It should be noted that the pathogenesis of plaque rupture and the consequence of acute complications of atherosclerosis (for example, ACS) are multi-factorial, as shown in Figure 6, and oxidative stress is only one of these factors.

### 2.1. Oxidative Stress in Cardiovascular Diseases: Mechanisms and Sources

Numerous data in the literature confirm that the presence of ROS plays an important role both at the stage of the initiation and maintenance of the atherosclerotic process. Potential sources of ROS in blood vessel cells include NADPH oxidase, respiratory chain, xanthine oxidase, nitric oxide synthase, cyclooxygenase, myeloperoxidases, and lipoxygenases [43].

#### 2.1.1. NADPH Oxidase (NOX)

NOX is present throughout the body. NOX consists of five subunits: membrane proteins gp91phox and p22phox, and cytoplasmic proteins p67phox, p47phox, and p40phox [44].

Seven isoforms of NADPH oxidase have been identified in human cells: NOX1, NOX2, NOX3, NOX4, NOX5, DUOX1, and DUOX2 [45]. NOX4 is a constitutively active enzyme and as a result of its action, H_2_O_2_ is formed. Other isoforms require activation and as a result of their action, the superoxide anion radical (O_2_^•−^) is generated. NOX is activated by the phosphorylation of the p47phox protein, and activating factors include cytokines, platelet-derived growth factor, epithelial growth factor, transforming growth factor beta 1 (TGF-β1), TNF-α, angiotensin II, endothelin, hypoxia, and mechanical factors (stretching, shear stress) [45]. The Rac protein is also needed to activate NOX [44,46].

In the vascular system, NOX1, NOX2, NOX4, and NOX5 seem to be important [44]. NOX activity is increased by atherosclerosis risk factors such as hypercholesterolemia, diabetes, obesity, metabolic syndrome, and hypertension [47]. It was found that human aortic endothelial cells subjected to hyperglycemia show increased NOX1 expression and increased ROS production, while the use of a NOX1 inhibitor reduces ROS production, the expression of adhesion molecules, and the infiltration of the vessel wall by inflammatory cells [48]. The role of NOX1 in neointimal proliferation after injury suggests its involvement in the early stages of atherosclerosis as well [49]. The involvement of NOX1 in vascular remodeling also involves inducing the proliferation, migration, and differentiation of vascular smooth muscle cells into macrophage-like cells, and these effects are related to the action of angiotensin II [50]. In turn, NOX2 is an important source of ROS in the endothelium, which results in the development of atherosclerotic lesions at an early stage and a reduction in the availability of nitric oxide [51].

In animal studies, it has been shown that NOX2 inhibition caused the regression of atherosclerosis in the aorta and a reduction in the expression of adhesion molecules and inflammatory cytokines [52]. NOX5, present in endothelial cells and macrophages, is involved in the development of atherosclerosis from the early stages [53]. It is believed that NOX4 mainly has protective properties consisting in inhibiting the proliferation of vascular smooth muscle cells and preventing endothelial damage, which helps maintain the endothelial barrier and increases the availability of nitric oxide [54,55]. On the other hand, there are some reports that indicate the involvement of NOX4 in the development of atherosclerosis [56].

Using animal models, it has been shown that ischemia increases the amount of mRNA encoding NADPH subunits as well as the amount of the protein itself in endothelial cells and lung epithelial cells [57,58], and also in cardiomyocytes [59]. However, even in the case of NADPH oxidase deficiency, in acute myocardial infarction, ROS are generated from other sources [59]. In a stroke model, the inhibition of NADPH oxidase activity during reperfusion results in a smaller area of brain necrosis and edema, as well as lower levels of oxidative stress markers [60]. In an animal model of ischemia and reperfusion, the deficit of NOX1 and NOX2, but not NOX4, had a protective effect on the heart: they reduced the infarct size and neutrophil infiltration. The same study showed that NOX1 and NOX2 are important, especially during the reperfusion period [61]. NOX2 activity is associated with adverse remodeling and left ventricular dysfunction after myocardial infarction [62].

#### 2.1.2. Respiratory Chain

Under physiological conditions, the mitochondrial respiratory chain (called also electron transport chain—ETC), in which the oxygen molecule undergoes a four-electron reduction, is a constant source of small amounts of the superoxide anion radical (O_2_^•−^). The electron transport chain is formed by four complexes, with approximately 1–2% of electrons “leaking” from it and being used to produce O_2_^•−^ [63]. The presence of superoxide dismutase (Mn-SOD) in the mitochondrial matrix and in the cell cytoplasm (Cu, Zn-SOD) allows the metabolism of the superoxide anion radical to form hydrogen peroxide, which has a longer half-life and the ability to penetrate biological membranes, and may act as a signaling molecule [64].

Under physiological conditions, the protonmotive force is a key regulator of the rate at which electrons flow through the ETC. The high proton gradient increases the energy cost of more protons moving into the intermembrane space, resulting in slower electron transport. The long residence time of electrons in the respiratory chain increases the probability of a one-electron reduction of O_2_ and the formation of a superoxide radical [65]. On the other hand, there are high-quality data showing that in the opposite situation, i.e., a low proton gradient, and also in a model that mimics ischemia, the mitochondria of cardiomyocytes also produce more ROS [66].

ETC uncoupling can also contribute to ROS production. Protonophores are small molecules that bind to the inner membrane of the mitochondria and allow protons to pass into the matrix, causing a mild uncoupling of the ETC. Reports so far indicate that although this may lead to increased ROS generation, it is rather a protective mechanism [67]. Uncoupling proteins (UCPs) are transmembrane transport proteins. Their activity is regulated by the presence of ROS on the basis of feedback—high levels of ROS activate UCPs, causing a decrease in the proton gradient and lower ROS production, so the action of UCPs inhibits oxidative stress. UCPs work most effectively at high proton gradients and require fatty acids to uncouple [68]. The mild uncoupling of oxidative phosphorylation also occurs with the activation of the ATP-sensitive K(+) channel. Both increased ROS production at low concentrations of ATP-sensitive K(+) channel activators [69] and decreased ROS production at high concentrations of ATP-sensitive K(+) channel activators have been reported [70]. The first situation is probably a mechanism involved in cardioprotection (preconditioning) [70], while the second may occur during the reperfusion period and reduce reperfusion damage [71].

The restoration of blood supply—reperfusion—results in increased ROS generation again. Complex I is the main source of ROS in reperfusion. The production of ROS by complex I in reperfusion can take place during forward electron transport and is caused by the fact that the complex I cofactor, flavin mononucleotide (FMN), remains in a reduced state, so that it cannot play the physiological role of electron acceptor [72]. The second mechanism of ROS generation is the result of reverse electron transport (RET) through complex I. Physiologically, the energy obtained from the transfer of two electrons from NAHD to CoQ is sufficient to pump four protons. In a situation where the proton gradient is high and there is a predominance of reduced CoQ, the energy obtained is not enough to transfer protons, and electrons are transported back. The predominance of reduced CoQ is significant for the period of ischemia and early reperfusion, and is associated with the accumulation of succinate, which is due to reverse succinate dehydrogenase activity. It is important to note that RET is the largest source of ROS in mitochondria [73,74].

Other mitochondrial mechanisms that contribute to ROS production during reperfusion include the increased activity of monoamine oxidase in the outer mitochondrial membrane [75], as well as the presence of NOX4, also located in the outer mitochondrial membrane [76]. Additionally, interactions between cytochrome c and p66Shc lead to the formation of hydrogen peroxide [77]. Another important process is ROS-induced ROS release (RIRR), which refers to the generation of a significant amount of ROS following a sudden increase in mitochondrial membrane permeability (mPTP), accompanied by a reduction in the proton gradient [78].

The mitochondrial permeability transition pore (mPTP) is a term used for the phenomenon of a sudden increase in the permeability of the intima of the mitochondrion, allowing the swollen movement between the matrix and the cytosol of particles weighing up to 1.5 KDa. The structure and components of mPTP are unknown, and as an attractive target for new forms of therapy, they are the subject of numerous hypotheses and studies [79]. The opening of the mPTP is considered to be the most important mechanism of reperfusion damage. In ongoing ischemia, anaerobic metabolism causes the accumulation of H^+^ ions in the cytosol and a decrease in pH. The H^+^/Na^+^ exchanger, trying to remove excess protons, causes the accumulation of sodium ions, which in turn activates the Na^+^/Ca^2+^ exchanger, which leads to Ca^2+^ overload. Acidosis inhibits the opening of the mPTP, while in reperfusion, the pH returns to normal. In this situation, factors such as the presence of Ca^2+^ overload, inorganic polyphosphate, ROS formation, and low level of nitric oxide lead to the opening of the mPTP [80]. The opening of the mPTP can be transient, serving as a protective mechanism by discharging excess calcium, inducting mild mitochondrial uncoupling, and regulating mitochondrial ROS release. The transient (rather than prolonged) opening of the mPTP is favored by a pH other than 7.4, the removal of Ca^2+^ ions or competition with other 2+ metal ions, and normal nitric oxide levels [81]. Reperfusion damage is associated with the prolonged opening of the mPTP. The prolonged opening of the mPTP results in the loss of the proton gradient and inhibition of oxidative phosphorylation. In such a situation, ATP synthase starts acting as ATPase, consuming ATP. The free migration of ions, in particular calcium, causes mitochondrial swelling and the damage and increased permeability of the outer membrane of the mitochondria [82]. This results in the release of cytochrome C, an intermembrane protein that is part of the respiratory chain, which, once in the cytoplasm, activates the apoptosis pathway. The Bax protein is responsible for increasing the permeability of the outer membrane of the mitochondria, enabling the release of cytochrome C. It is also proposed (together with the Bak protein) as a component of the mPTP [83]. In summary, oxidative stress and mPTP opening are considered to be the most important components of reperfusion injury, and the inhibition of both processes results in protection against damage [84]. Moreover, the overproduction of ROS in the mitochondria contributes to the increased biosynthesis of pro-inflammatory cytokines, which promotes the formation of atherosclerotic plaque [85]. Furthermore, at the early stage of atherosclerosis development, ROS generated in mitochondria through lysophosphatidylcholine causes dysfunction of the vascular endothelium [86]. In addition, oxidative damage to mitochondrial DNA reduces the efficiency of the cellular respiration process, which in turn increases the apoptosis of macrophages and vascular smooth muscle cells and leads to an enlargement of the necrotic core of the atherosclerotic plaque and thinning of fibrous cap [87]. Mitochondrial DNA is very susceptible to oxidative damage, which results in the increased progression of atherosclerosis [88], and an elevated level of mitochondrial DNA in the circulation is a reliable indicator of the risk of death within 30 days after a myocardial infarction [89]. It is also suggested that the increased activity of NOX4 observed in the course of atherosclerosis increases the production of ROS in mitochondria [90].

Mitochondrial oxidative stress in immune cells has also been shown to promote atherosclerosis. In neutrophils, it is associated with the increased secretion of neutrophil extracellular traps (conglomerates of fibers, proteins, and mitochondrial DNA) [91], while in macrophages it is due to the intensification of the pro-inflammatory NF-κB pathway, chemotaxis, and the penetration of monocytes into the atherosclerotic plaque [92].

#### 2.1.3. Nitric Oxide Synthase (NOS)

Nitric oxide (NO) is the most important factor in the vascular system providing vasodilatation, antiaggregation, and anti-inflammatory effects of the endothelium. The reaction of NO production is catalyzed by NOS using L-arginine as a substrate. There are three isoforms of NOS in the circulatory system: neuronal (nNOS, especially in cardiomyocytes), inducible (iNOS, in macrophages/monocytes, neutrophils, endothelium, vascular smooth muscle), and endothelial (eNOS, in endothelium, lymphocytes, neutrophils, eosinophils) [93].

**Figure 7:**
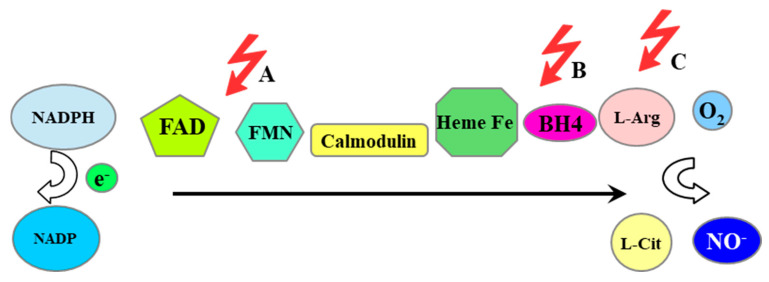
Mechanism of action of NOS and potential uncoupling sites. The red arrows mark the places of potential uncoupling of the NOS: A. S-glutathionylation prevents the transport of an electron from FAD to FMN, and the electron is transferred directly to O_2_ to form a superoxide anion radical. B. Deficiency/oxidation of BH4 disrupts the flow of the electron, which is transferred to O_2_ without the use of arginine. C. L-arginine deficiency results in oxygen being the electron acceptor.

NADPH is involved in the action of NOS. NADPH is an electron donor transferred to subsequent cofactors and finally to heme (Figure 7). Another electron from tetrahydrobiopterin (BH4) is transferred to oxygen. The effect of NOS is the formation of nitric oxide and citrulline.

Critical for the proper functioning of NOS is the availability of the cofactor BH4 and arginine as a substrate. The main factors limiting the availability of BH4 include oxidative stress, angiotensin II, and homocysteine [94]. The availability of arginine is limited by cytoplasmic arginase I and mitochondrial arginase II, enzymes that metabolize arginine to urea and L-ornithine, as well as the competitive arginine inhibitor dimethylated arginine (ADMA). The action of arginase II is therefore an additional factor regulating the production of ROS by NOS [95]. The lack of factors critical for the functioning of NOS causes its uncoupling and the production of superoxide anion radical instead of NO [93]. Thus, a deficiency of L-arginine as a substrate blocks the production of NO, and the mobilized electron is transferred to O_2_ [96]. The decoupling of NOS also causes S-glutathionylation by attaching oxidized glutathione to the thiol group of cysteine. S-glutathionylation occurs at the binding site of the NOS cofactors, flavin adenine dinucleotide (FAD) and flavin mononucleotide (FMN), which disrupts electron transport, and in this situation, the electron is transferred to oxygen and a superoxide anion radical is formed [97]. Nitric oxide generated by the NOS is the most important factor responsible for the proper function of the endothelium. The uncoupling of the NOS therefore causes nitric oxide deficiency and the loss of physiological properties of the endothelium. This endothelial dysfunction, which arises due to NOS uncoupling and the resultant oxidative stress, is a key contributor to the development of ischemic heart disease. However, as a result of the reaction of superoxide anion with NO, the NO pool is reduced and at the same time a very reactive peroxynitrite is formed, which disturbs the redox balance.

Risk factors for atherosclerosis such as hypertension, hypercholesterolemia, nicotinism, and type 2 diabetes are characterized by reduced levels of NO [98]. Thus, both NO release and eNOS expression are reduced in the endothelium of the arteries affected by atherosclerotic lesions [99]. The inhibition of arginase activity results in many benefits, for example an increase in NO production, improved endothelial function, and a reduction in the severity of atherosclerosis [100]. Similarly, the supply of an additional BH4 pool results in increased NO production, decreased production of superoxide anion radicals, inhibition of the progression of atherosclerosis, and reduced infiltration of the vessel wall by leukocytes [101]. As a consequence, in experimental ischaemia–reperfusion models, both nNOS and eNOS exhibit protective effects and improve the prognosis after an ischemic episode [102,103]. On the other hand, high iNOS activity is associated with the progression of atherosclerosis [104], the occurrence of angina, and thrombus on atherosclerotic plaque, as well as the presence of nitrotyrosine—a protein marker of oxidative stress [105]. In addition, based on the results of studies obtained in animal models, the high expression of iNOS was observed in the myocardium of patients with myocardial infarction [106], which is associated with the severity of myocardial damage in the course of ischemia and reperfusion [107]. Additionally, in patients who qualified for CABG (coronary artery bypass grafting), it was shown that the increased level of dimethylated arginine in the blood serum was correlated with an increased amount of superoxide anion radical in the wall of the saphenous vein and internal mammary artery and less relaxation in response to acetylcholine, while the activity of NADPH remained unchanged [108].

#### 2.1.4. Xanthine Oxidase

Another pro-oxidant enzyme is xanthine oxidase (XO), which is responsible for the final stage of purine transformations, catalyzing the reaction of the conversion of hypoxanthine into xanthine and then into uric acid (UA), producing the superoxide anion radical or hydrogen peroxide. Uric acid is an important plasma antioxidant that can remove, among others, the very reactive peroxynitrate (ONOO^−^). On the other hand, UA can inhibit NO production, induce inflammation, and participate in reactions leading to the generation of ROS [109]. It has also been shown that an elevated uric acid level is an independent risk factor for atherosclerosis [110]. Consequently, the potentially proatherogenic effects of XO are thought to be due to the production of ROS and uric acid.

High XO activity is associated with the presence of risk factors for coronary heart disease, such as hypertension [111], chronic kidney disease [112], diabetes [113], obesity [114], and smoking [115]. In addition, increased plasma XO activity is associated with the presence of more advanced atherosclerotic lesions in the coronary arteries [116]. It has also been shown that the use of allopurinol (XO inhibitor) inhibits the transformation of macrophages into foam cells and the production of inflammatory cytokines, adhesion molecules, and metalloproteinases in already formed foam cells [117]. Increased XO activity was also found in macrophages extracted from atherosclerotic plaque and in the aortic endothelium. In the same study, after the use of an XO inhibitor, a reduction in ROS production and the inhibition of the progression of atherosclerosis were observed [118]. Atherosclerotic plaques collected from the carotid arteries of patients showing symptoms of ischemia were also characterized by a higher expression of the XO protein compared to plaques from asymptomatic patients, which indicates a significant role of XO in the process of plaque destabilization [119]. As a consequence, it has been proposed to use the measurement of XO activity as a simple method for detecting acute atherosclerosis complications, e.g., diagnosing myocardial infarction [120]. In a small group of patients, it has been shown that the use of allopurinol in myocardial infarction improves the short-term prognosis [121]. In addition, the inhibition of XO activity in the acute phase of myocardial infarction prevented the occurrence of abnormalities in skeletal muscle and exercise intolerance [122]. Based on meta-analyses carried out in 2018 and 2020, it was shown that XO inhibition has a protective effect in the secondary prevention of myocardial infarction, major adverse cardiovascular events (MACEs), and an endpoint defined as total cardiovascular events, while in the entire study population, these effects were not statistically significant [123,124].

### 2.2. Antioxidant Defense Mechanisms in the Circulatory System

**Figure 8:**
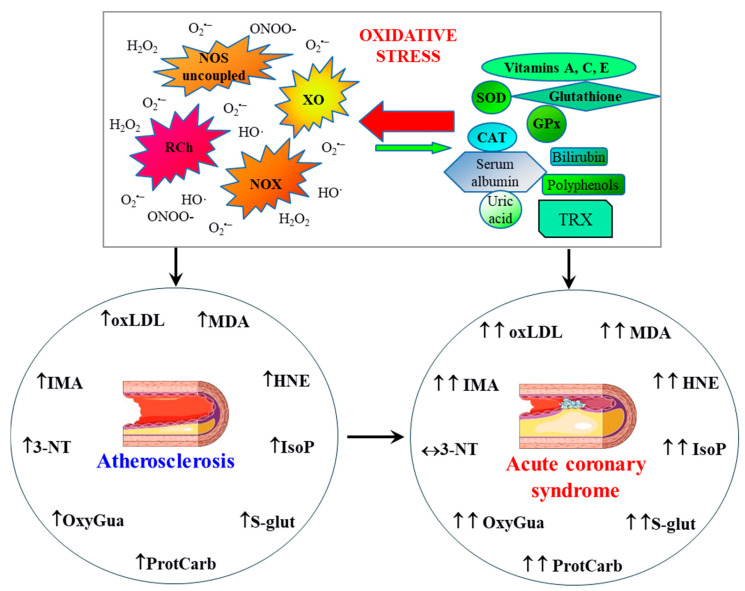
Oxidative stress in atherosclerosis and ACS. Oxidatively modified guanine (GuaOxy), ischemia-modified albumin (IMA), nitric oxide synthase (NOS), protein carbonyl (ProtCarb), respiratory chain (RCh), S-glutathionylation (S-glut), thioredoxin (TRX), and 3-nitrotyrosine (3-NT). ↑ level increase; ↓ level decrease; ↔ no change in the level.

Taking into account that the formation of pro-oxidant conditions plays an important role in initiating and maintaining the atherosclerotic process, an important protective element is providing the body with adequate antioxidant protection at the level of proteins and enzymes (Figure 8). However, it has been found that the total antioxidant status (TAS) in patients with angiographically confirmed CAD is lower than in the general population [125]. In addition, Bastani and colleagues showed that the TAS value correlates negatively with the number of vessels involved in stable CAD [126].

Among the enzymes that provide antioxidant protection to blood vessel cells, classic antioxidant enzymes, including SOD, GPx, and CAT, play a key role. However, data on changes in the activity of these enzymes in CAD are very divergent and probably depend on the stage of the disease. A decrease in SOD was observed in people at high risk of CAD, which was also associated with high platelet activity during acetylsalicylic acid treatment [127]. In patients diagnosed with CAD, compared to healthy controls, in erythrocytes, the reduced activity of SOD and CAT were observed, with no differences in GPx activity [128]. This correlates with reduced mRNA expression for SOD in lymphocytes and peripheral blood monocytes from patients with CAD [125]. However, the above data are contradicted by the results of studies in which an increase in SOD activity in chronic CAD was found, probably related to the pro-oxidative and proatherogenic effects of SOD at high concentrations [129], which was associated with a worse outcome [130]. In another study, elevated SOD and CAT levels were found only in the early stages of CAD in response to oxidative stress, while the activity of both enzymes was reduced in the more advanced stages of the disease [131]. In addition, a negative correlation was found for the GSH-dependent antioxidant system, including glutathione levels and GPx activity in association with the severity of stable CAD, with significantly reduced GPx activity found in ACS [126]. It should be emphasized that there are also data proving that there is no relationship between changes in the activity of antioxidant enzymes and the risk of occurrence or the severity of CAD. SOD, GPx, and CAT levels in erythrocytes of women who were generally healthy at the time of blood collection were not associated with future CAD [132].

In contrast, in ACS, there is a consensus that there is an imbalance between ROS generation and elimination. In the entire study group from the above-cited study [126], the lowest TAS level was observed in ACS, and intermediate TAS level was observed in stable CAD. In addition, in one of the more recent studies in patients with NSTEMI, among many antioxidant parameters, only the TAS value had a prognostic significance for predicting long-term mortality [133]. In a study of young people (age < 35 years) diagnosed with myocardial infarction, TAS was reduced compared to controls, but did not correlate with the severity of CAD [134]. This correlates with an increase in the level of TAS and other parameters, indicating an improvement in antioxidant defense in patients undergoing cardiac rehabilitation [135].

Most studies indicate reduced SOD and CAT activity in the course of ACS [136,137]. However, in unstable angina pectoris, increased SOD and CAT activity is observed in blood from the coronary artery and increased SOD activity is observed in peripheral venous blood [138]. It is also believed that low SOD activity may be an independent predictor of the occurrence of cardiovascular events in the future [139,140]. However, there are also reports of both elevated [141] and decreased [142] GPx activity in ACS. In contrast, it was shown that higher SOD and GPx levels in STEMI correlated with a faster resolution of ST-segment elevation, the return of a full flow in coronary arteries after coronary angiography, and a less frequent occurrence of heart failure [143]. In a study conducted by Holley and colleagues evaluating the predictive value of GPx and SOD activity in ACS, there was no association with the occurrence of cardiovascular events in the following 12 months [144]. In a later study, the same group of researchers found an inverse correlation between GPx activity in ACS and the occurrence of cardiovascular events at a 12-month follow-up period [145].

## 3. Oxidative Modifications and Their Consequences in Atherosclerosis and ACS

### 3.1. Lipid Peroxidation

Increased pro-oxidative processes and the impairment of antioxidant defense mechanisms in the course of CAD cause a shift in the redox balance towards oxidative processes. This is evidenced by data in the literature confirming the increase in oxidative modifications of the most important cell components: lipids, proteins, and nucleic acids. Oxidative lipid modifications are of key importance in the development of atherosclerosis and, consequently, CAD [146]. As a consequence, changes in cellular structures, especially cell membranes, initiate a number of processes contributing to the progression of the disease. Therefore, understanding these mechanisms is crucial to understanding the causes and consequences of atherosclerosis and developing more effective treatment methods. Among lipid compounds, the most susceptible to oxidation are both low-density lipoproteins (LDLs) and components of cell membrane phospholipids: polyunsaturated fatty acids (PUFAs).

Oxidized phospholipids have been shown to change the endothelial phenotype into pro-inflammatory and pro-aggregation types by regulating gene expression [147]. As a result of the free radical peroxidation of PUFAs, lipid peroxides are formed, which undergo further transformations to produce final products in the form of low molecular weight unsaturated aldehydes such as MDA, 4-hydroxy-2-nonenol (HNE), and IsoP (Figure 8), which, through modifications in the structure and function of proteins, show regulatory properties to endothelial cells [148]. Elevated levels of MDA were found in patients with the presence of risk factors for the development of atherosclerosis, such as hypertension [149], obesity [150], hyperlipidemia [151], and smoking [152]. Moreover, the PREVENT study showed that the level of MDA in the plasma of patients with stable CAD increases with the progression of atherosclerotic lesions and the number of coronary arteries affected by atherosclerosis. Therefore, MDA has been found to be a predictor of cardiovascular events independent of traditional risk factors [153]. HNE, a product of the oxidation of polyunsaturated omega-6 fatty acids, due to its high chemical reactivity, can directly react mainly with thiol groups of proteins, modifying their structure and function and, consequently, leading to concentration-dependent modifications of numerous signaling pathways [154]. As a result, the involvement of HNE in the formation of atherosclerosis is found at every stage of this process, from the oxidative modification of proteins and lipoproteins, and then through inflammation, the migration of inflammatory cells and vascular smooth muscles, to apoptosis and neovascularization [154,155].

In turn, IsoP, which are products of the oxidative cyclization of PUFAs, have an effect that enhances platelet aggregation, monocyte adhesion to the endothelium, vasoconstriction, angiogenesis, and pro-inflammatory effects [156]. It has been shown that the presence of classic risk factors for CAD, such as obesity, smoking, hyperlipidemia, and diabetes, is accompanied by an approximately 2–3-fold increase in the concentration of IsoP in the blood serum [157]. Elevated IsoP levels were also observed in the urine of young, healthy individuals with calcifications in the coronary arteries, which confirms the involvement of oxidative stress in the early stages of atherosclerosis [158]. It was also found that a significantly elevated plasma IsoP content is an independent predictor of peripheral atherosclerosis [159]. In addition, the presence of angiographically significant atherosclerotic lesions in the coronary arteries is associated with higher levels of IsoP [160]. Moreover, it has been shown that an increase in the content of the most recognized oxidative stress biomarker among all IsoP, 8-isoprostaglandin F(2alpha) (8-iso-PGF_2α_) in blood serum, correlates with the number of coronary artery segments affected by atherosclerotic lesions [161], and a similar relationship was observed for the amount of IsoP excreted in the urine [162]. Consequently, increased urinary excretion of 8-iso-PGF_2α_ in patients with diagnosed CAD was considered an additional risk factor [163]. Interestingly, the determination of the IsoP level before coronary artery bypass grafting allows for the prediction of an increased risk of death from cardiovascular causes in long-term follow-ups (mean follow-up 11.7 years) [164]. Additionally, a high excretion of 8-iso-PGF2_2α_ in the urine of postmenopausal women has been found to increase the risk of death from CAD or stroke by 80% [165].

In addition to the lipid derivatives mentioned above, the interaction of ROS with LDL produces oxidized LDL (oxLDL), which, due to its participation in numerous processes shown in Figure 8, leading to the development of atherosclerosis, has been considered a useful marker of cardiovascular diseases. The level of oxLDL in the blood has a predictive value for the occurrence of subclinical carotid atherosclerosis [166], cardiovascular events [167], and multiple endpoints (CV events: cardiac death, MI, PTCA, and ischemic stroke) [168]. Elevated oxLDL levels in healthy subjects were found both in plasma and in carotid plaques in patients with carotid atherosclerosis [169]. It has also been shown that antibodies to oxLDL correspond to the occurrence and severity of CAD [170]. The oxLDL to LDL ratio may also be a useful marker of coronary atherosclerosis severity in patients with type 2 diabetes in clinical practice [171]. At the same time, it should be emphasized that, in addition to the above-mentioned studies, there are also few reports indicating that elevated oxLDL levels are only associated with low-grade CAD [172], as well as the lack of significant differences in the level of anti-oxLDL antibodies regardless of the severity of the CAD [173], and studies questioning the predictive value of oxLDL for the occurrence of cardiovascular events in patients with diagnosed CAD [174]. Moreover, it is also possible that IgM [175] and IgG [176] anti-ox-LDL antibodies have a protective effect on the occurrence of cardiovascular events.

It has been shown that the development of atherosclerosis is associated with an increased level of oxidized lipids assessed by the level of oxLDL in the blood serum, while a further increase is observed in the group of patients with unstable angina and an even higher level in patients with myocardial infarction [177]. Significantly more oxLDL-containing macrophages were also found in atherosclerotic plaques collected from the coronary arteries of patients with unstable angina compared to patients with stable CAD [177]. In ACS, higher levels of antibodies to oxLDL were also found compared to patients with chronic CAD [178]. Both the level of oxLDL and anti-oxLDL antibodies in ST segment elevation infarction was higher compared to the control and increased with the number of arteries with significant atherosclerotic lesions [179]. Consequently, it is believed that oxLDL may be a marker of the severity of the disease and outcome in patients after a myocardial infraction [180].

However, regardless of the possibility of using oxLDL in the diagnostic and therapeutic process, other lipid peroxidation products are also increasingly used in the assessment of the development of atherosclerosis and severity of ACS. In myocardial infarction, an increased level of MDA and its correlation with the patient’s condition assessed according to clinical criteria, e.g., on the GRACE scale, have been demonstrated [181]. On the other hand, during the 3-year follow-up, it was found that after ACSs, there is a decrease in MDA levels, which is interpreted as an effect of the restoration of the antioxidant barrier [182]. In addition, it was found that the level of another marker of lipid oxidation, HNE, in patients with myocardial infarction, is elevated and correlates with the level of troponin and NTproBNP, as well as with the size of the infarction [183]. It is also believed that HNE may contribute to atherosclerotic plaque instability by activating metalloproteinase 9 [184].

In ACS, significant changes in the level of IsoP in a variety of material from patients are also observed. The level of 8-iso-PGF_2α_ is significantly higher in atherosclerotic plaques collected from unstable patients, which indicates the involvement of oxidative stress and IsoP in the formation of unstable plaques [185]. Moreover, it was found that in patients with infarction, the level of IsoP is higher and increases within an hour after coronary angiography and tends to decrease after six hours. In the same study, it was also observed that a longer time from the onset of pain to the PCI (percutaneous coronary intervention) of the culprit lesion was associated with higher IsoP levels in samples taken one hour after coronary angiography [186]. In addition, clots collected from patients in ACS were characterized by a greater durability and resistance to fibrinolysis compared to material collected in chronic coronary syndromes, and the predictive factors were the levels of 8-iso-PGF_2α_ and CRP [187]. Another study observed that elevated levels of 8-iso-PGF_2α_ in ACS (compared to stable coronary syndromes) was correlated with elevated levels of markers of platelet activation [188]. The transient increase in oxidative stress as a result of angioplasty in ACS, assessed by an increase in IsoP levels, confirms the results obtained from both blood and urine samples [186,189]. IsoP levels are also thought to have predictive value, as they can predict the 30-day risk of cardiovascular events after myocardial infarction [190].

### 3.2. Protein Oxidation

**Figure 9:**
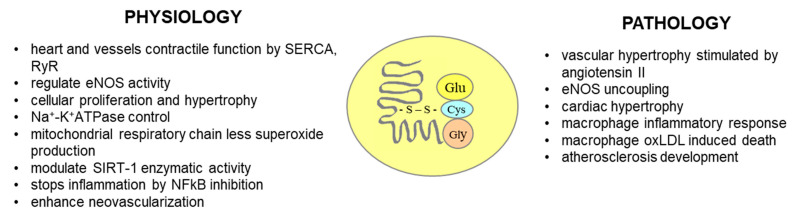
Physiological and pathological role of S-glutathionylation in circulatory system. SERCA—sarco/endoplasmic reticulum Ca^2+^-ATPase; RyR—ryanodine receptor; eNOS—endothelial nitric oxide synthase; Na^+^-K^+^ATPase—sodium/potassium pump; SIRT-1—sirtuin 1; NFkB—nuclear factor kappa B; Glu—glutaminic acid; Cys—cysteine; and Gly—glycine.

Oxidative modifications of macromolecular compounds of the human body concern not only lipids, but also proteins. The most studied and described markers of the oxidative modification of proteins associated with CAD are S-glutathionylated proteins and 3-nitrotyrosine (Figure 9). S-glutathionylation is a physiological mechanism of the post-translational modification of proteins, consisting in the formation of disulfide bridges between cysteine glutathione and protein residues. Increased levels of proteins modified by S-glutathionylation are observed under oxidative stress conditions [191]. S-glutathionylation was found to be increased in oxLDL-treated macrophages and is one of the factors inducing macrophage death [192]. The S-glutathionylation of the eNOS protein causes its uncoupling [97]. In addition, increased levels of proteins modified as a result of S-glutathionylation were found in the plasma of patients with peripheral atherosclerosis [193]; moreover, the observed increase was correlated with the severity of atherosclerosis measured by the ankle–brachial index [193]. The severity of S-glutathionylation was studied in patients undergoing coronary artery bypass grafting (CABG) who were simultaneously undergoing aortic valve replacement. An increased content of proteins modified as a result of S-glutathionylation was found on valves affected by atherosclerotic lesions compared to valves without atherosclerotic lesions [194]. It is known from studies on animal models that S-gluathionylation may play a role in the myocardial response to ischemia, because S-glutathionylation increases the release of calcium from the sarcoplasmic reticulum by modifying the ryanodine receptor [195] and increasing the force of heart muscle contraction. Additionally, it stimulates sarcoplasmic reticulum calcium ATPase (SERCA) to accelerate calcium uptake, causing faster and more effective relaxation [196].

Another marker of protein modification that plays an important role in CAD is 3-nitrotyrosine (3-NT). 3-NT is formed as a result of the reaction of reactive nitrogen species, primarily including peroxynitrite (ONOO^−^), produced by the reaction of nitric oxide (II) with the superoxide anion radical, as well as nitric (IV) oxide (NO_2_) (formed as a result of the reaction of •NO with hydrogen peroxide) with tyrosine [197]. It was found that as a result of the tyrosine nitration of prostacyclin synthase, the activity of this enzyme is inhibited, which results in a decrease in the level of prostacyclin—the most important anti-atherogenic prostanoid [198]. Moreover, subjecting fibrinogen to ONOO significantly accelerates clot formation [199].

The presence of 3-NT was found in atherosclerotic plaques removed from the carotid arteries of patients with stable CAD [200]. Moreover, in the blood serum of these patients, the level of antibodies against protein-bound nitrotyrosine was 10 times higher compared to healthy subjects and was positively correlated with the severity of atherosclerotic lesions assessed angiographically [200]. Increased levels of both free 3-NT and myeloperoxidase were found in the blood serum of patients with CAD compared to healthy people [201]. In addition, nitrotyrosine has been shown to promote the proliferation and metabolic activity of vascular smooth muscle cells [202].

Important products of protein modification also include protein carbonyl groups (PC), which are the result of the oxidative modification of amino acids, most often proline, arginine, lysine, and threonine. Modification can occur by direct reaction with a free radical or with a reactive aldehyde such as HNE [203,204]. Familial hypercholesterolemia, both homo- and heterozygous, is associated with elevated serum PC levels [205]. In atherosclerotic plaques collected from the carotid arteries, an increased level of PC was found in relation to sections of a healthy artery, but the level of PC did not distinguish between symptomatic and asymptomatic patients, or stable and unstable plaques [206]. In CAD, elevated levels of PC in the blood were also found, which correlated with other markers of oxidative stress [207,208]. However, the predictive value of PC is uncertain, as the concentration of serum carbonyl groups does not allow for the prediction of the risk of MACE in a 64-month follow-up in patients with type 2 diabetes [209], but the level of LDL-associated PC can be used to distinguish between healthy and CAD patients [210].

The most common proteins in the human blood are albumin, which constitute about 40% of plasma proteins, and whose N-terminal fragment is very susceptible to modification by ROS, which reduces the affinity of the protein for transition metal ions, in particular for cobalt ion (Co^2+^). Albumin oxidatively modified in this way is called ischemia-modified albumin (IMA) [211]. The concentration of IMA shows a positive correlation with the thickness of the intima–media complex and has been proposed as a marker for the initial stages of atherosclerosis [212]. Linking IMA levels to the thickness of epicardial adipose tissue may be an alternative method of the non-invasive evaluation of CAD [213]. The IMA level is significantly elevated in angiographically confirmed CAD and correlates with the number of diseased coronary arteries [214]. Measuring the level of IMA in the population of patients undergoing continuous ambulatory peritoneal dialysis (CAPD) allows for an effective assessment of the risk of cardiovascular events [215].

In ACS, especially in the initial period of reperfusion, increased S-glutathionylation occurs. 3-phosphoglyceraldehyde dehydrogenase (GAPDH), an enzyme involved in glycolysis, is one of the most important proteins undergoing S-glutathionylation in acute ischemia. It is therefore believed that the S-glutathionylation of GAPDH may be a protective mechanism by preventing the irreversible modification of this enzyme. However, even the transient loss of GAPDH activity may contribute to ischemic cell damage, and GAPDH modification may promote apoptosis pathways [216]. It is also known that the S-glutathionylation of G-actin hinders its polymerization and connection with myosin, which impairs myocardial contractility in the course of ischemia [217]. Moreover, the S-glutathionylation of the mitochondrial respiratory chain complex I inhibits electron transport and causes the production of the superoxide anion radical [218].

In clots aspirated from patients with STEMI, a correlation was found between the concentration of 3-NT and the level of inflammatory parameters in the clot [219]. On the other hand, the level of 3-NT in the blood measured several times in the course of STEMI did not differ from the level in the control group [186]. Moreover, there are reports, based on a 4-year follow-up of patients with acute coronary syndrome in the ERICO study, in which no correlation was found between serum 3-NT levels and mortality in patients with acute coronary syndrome [220,221].

It has also been shown that the concentration of carbonyl groups (PC) is significantly elevated in myocardial infarction, both STEMI and NSTEMI [222]. In acute myocardial infarction, PC and IMA levels allowed good discrimination between healthy and ill patients, and the combination of these two markers showed high sensitivity and specificity in diagnosing infarction [223]. Similarly, the high sensitivity and specificity of PC and IMA were found in another study [222], in which the study group included patients with different forms of ACS: UA, STIMI, and NSTEMI. Additionally, it has been shown that in patients after a myocardial infarction, the formation of carbonyl groups concerns fibrinogen in particular. Modified in this way, fibrinogen shows faster polymerization, which results in easier clot formation [224].

IMA levels increase significantly in ACS. An increase in IMA levels can be found as early as 6–10 min after the onset of ischemia [225], with IMA levels significantly increasing in STEMI, NSTEMI, and unstable angina, but it does not distinguish between these three conditions. An increase in IMA levels occurs earlier than troponin levels [225,226]. Importantly, measuring the level of IMA allows for distinguishing ACS from non-cardiac causes of chest pain [227]. Additionally, measuring the level of several markers at the same time, e.g., IMA+/− troponin+/− CK MB Mass correlated with ECG recording, additionally improves the sensitivity and specificity of ACS diagnosis [228]. In NSTEMI, IMA levels correlate with the extent of atherosclerotic lesions in the coronary arteries [229]; in turn, in STEMI, it can be used as a marker of persistent ischemia despite the use of reperfusion therapy [230]. The determination of IMA levels within 24 h of admission in patients with myocardial infarction is an independent prognostic factor in a 1-year follow-up [231]. The IMA assay (Albumin Cobalt Binding—ACB) was approved by the FDA in 2003 to exclude myocardial infarction [232]. However, due to numerous objections regarding the ACB method itself, as well as the specificity and diagnostic value of IMA, the IMA test is currently not used in clinical practice [233].

### 3.3. DNA Oxidation

Oxidative stress accompanying the development of atherosclerosis also affects the intensification of the generation of oxidative modification products of nucleic acids. Among the nitrogenous bases, guanine is particularly susceptible to oxidative modifications. The product of such reactions is 8-hydroxy-2-deoxyguanosine (8-OHdG) and 8-oxo-7,8-dihydroguanosine (8-oxoGuo) [234]. Increased levels of oxidatively modified guanine products and the increased activity of DNA repair systems were found in atherosclerotic plaques removed from carotid arteries compared to cells of a healthy internal carotid membrane and a section of a healthy internal thoracic artery [235]. In addition, 8-OHdG levels have been shown to correlate with the progression of atherosclerotic lesions in the coronary arteries [236]. It has also been found that the accumulation of 8-oxoGuo in vascular smooth muscle cells is associated with the reduced activity of the repair enzyme OGG1 (8-oxoGuo DNA glycosylase 1). In addition, studies conducted on mice have shown that higher levels of OGG1 significantly reduce the development of atherosclerosis [237]. The level of 8-OHdG in the mitochondrial DNA (mtDNA) of diabetic patients with a 50% stenosis of at least one coronary artery was significantly elevated and correlated with the severity of CAD. Moreover, as a result of a one-year follow-up of patients who underwent revascularization, the predictive value of the 8-OHdG level was confirmed for the multiple endpoints: MACCE (major adverse cardiovascular and cerebral events), death from any cause, and cardiac death [238]. Based on a six-year follow-up, it was shown that the concentration of urinary excreted 8-oxoGuo in diabetic patients correlated with both all-cause mortality and the occurrence of cardiovascular diseases [239]. A similar relationship was observed in a group of almost 10,000 people from the general population in Germany [240]. Also, the analysis of the results of 14 studies confirmed the association of elevated levels of 8-OHdG in urine and blood with cardiovascular diseases caused by atherosclerosis [241]. In contrast, in patients with CAD diagnosed using angiography, a lower level of 8-OHdG is associated with the presence of a “more favorable” allele in the p22phox subunit gene of NADPH oxidase and, as a result, a lower frequency of MACE in long-term follow-up [242].

In peripheral blood lymphocytes in patients with ACS, a higher incidence of DNA damage was found than in the group of healthy controls, and in addition, the amount of damage in the group with myocardial infarction is higher than in unstable angina [243]. In addition, it was found that higher plasma levels of 8-OHdG in patients with myocardial infarction correlate with a risk assessment based on clinical parameters [181]. Mitochondrial DNA (mtDNA) has been shown to be damaged, especially in the early phase of reperfusion, while OGG1 activity plays an important role in mtDNA repair in ischemia and reperfusion [244]. Reperfusion therapy in myocardial infarction lowers plasma levels of 8-OHdG, with a greater reduction observed with coronary angioplasty than with thrombolysis [245]. The assessment of the level of 8-OHdG in the urine of patients with acute coronary syndrome is considered to be a good predictor of cardiovascular death within the next 34 months [246]. In addition, high levels of guanosine oxidation products in DNA (8-OHdG and 8-hydroxyguanine) and RNA (8-hydroxyguanosine and 8-hydroxyguanine) are associated with a worse prognosis in patients with myocardial infarction complicated by cardiogenic shock [247]. High-dose atorvastatin treatment in ischemic cardiomyopathy was associated with a significant decrease in the plasma 8-OHdG level, which was correlated with an improved left ventricular systolic function [248].

The above-mentioned data regarding changes in the level/activity of selected antioxidants and the content of markers of oxidative modifications of lipids, proteins, and nucleic acids in atherosclerosis and ACS are summarized and presented schematically in Table 4.

## 4. Antioxidants and Their Biomedical Value Atherosclerosis and CAD

As mentioned earlier, ROS can induce endothelial dysfunction, promote lipid oxidation, and enhance inflammatory responses, which are key processes in atherosclerosis and plaque instability. Over the past 15–20 years, various therapeutic approaches have been proposed for treating atherosclerosis and coronary heart disease, focusing on drugs with antioxidant properties. These therapies include statins, antiplatelet drugs, beta-blockers, angiotensin-converting enzyme inhibitors, and angiotensin receptor antagonists [258,259].

Statins are the basis of therapy for all types of atherosclerosis [260]. In addition to lowering lipid levels, statins also have anti-inflammatory and antioxidant effects. Statin therapy has been shown to increase blood levels of the antioxidant enzymes GPx and SOD, but does not affect CAT levels [261]. Additionally, statins activate the Nrf-2/HO-1 pathway, enhancing antioxidant effectiveness and providing increased protection against oxidative stress [262]. Statins also inhibit the activity of NADPH oxidase [263] and protect lipids from peroxidation, although they do not prevent oxidative modifications of proteins and nucleic acids [264].

While statins are a key element in the treatment of atherosclerosis, another group of drugs particularly recommended for CAD is antiplatelet drugs, including acetylsalicylic acid. Like statins, acetylsalicylic acid activates the Nrf-2/HO-1 pathway [265] and also inhibits the pro-inflammatory NF-κB pathway [266]. Studies have shown that aspirin positively affects the expression of antioxidant enzymes, including SOD, CAT, and GPx [267]. However, the administration of low doses of aspirin to both healthy volunteers [268] and patients with CAD [269] has been found to reduce markers of oxidative stress, including oxLDL, IsoP, and total oxidative status. Additionally, inhibitors of the P2Y12 receptor, including clopidogrel, ticagrelor, and prasugrel, have been shown to be critical in the treatment of myocardial infarction and for patients following coronary angioplasty [270]. Animal studies indicated that both clopidogrel and ticagrelor exhibit antioxidant properties, as indicated by reduced levels of oxidative stress markers [271,272]. Clinical studies have also shown reduced levels of these markers in patients treated with these medications [273,274,275]. Additionally, it is worth noting that ticagrelor and prasugrel may offer greater antioxidant benefits compared to clopidogrel [276].

Another group of drugs recommended for the treatment of coronary heart disease is beta-adrenergic receptor antagonists, or beta-blockers. Among the members of this group, carvedilol is notable for its ability to regulate the generation and capture of ROS, which enhances its antioxidant properties [277]. Moreover, it is known that in chronic CAD, the activity of antioxidant enzymes such as SOD, GPx, and CAT is lower compared to that in healthy individuals. Carvedilol therapy increases the activity of these enzymes, with GPx activity reaching levels observed in healthy volunteers [278]. Additionally, carvedilol inhibits the production of ROS by leukocytes and protects amino acids from oxidative damage [279]. It also inhibits the activity of metalloproteinases 2 and 9 [280] and provides mitochondrial protection by inhibiting the mitochondrial permeability transition (MPT) [281]. An effect comparable to that of carvedilol has also been demonstrated with nebivolol [282], which reduces ROS production in endothelial cells, lowers oxidative stress markers, and increases the availability of nitric oxide, preventing its oxidative degradation [283,284]. Moreover, another beta-adrenergic receptor antagonist, metoprolol, has been found to have a stronger antioxidant effect compared to carvedilol and nebivolol [285].

Antioxidant properties are also exhibited by angiotensin-converting enzyme inhibitors (ACE-Is) and angiotensin receptor antagonists (ARBs), which are commonly used in cardiovascular diseases [286]. Captopril, due to its -SH groups, is recognized for its potential antioxidant properties. It was among the first ACE inhibitors shown to have antioxidant activity in vitro, although other ACE inhibitors, such as enalapril and lisinopril, have also demonstrated similar effects [287,288]. Zofenopril, a newer ACE inhibitor, also has the ability to reduce oxidative stress in animal models by decreasing the expression of NADPH oxidase and increasing GPx activity, which consequently leads to a reduction in the levels of protein carbonyl groups [289]. Patients treated with zofenopril had lower levels of lipid peroxidation products, such as MDA and IsoP, and better bioavailability of nitric oxide compared to those treated with enalapril [290]. The antioxidant effect of zofenopril has also been confirmed by randomized studies involving small groups of patients [291,292], which demonstrated an association between improvements in oxidative stress parameters and a slowdown in the progression of atherosclerosis [291]. However, in animal studies, losartan demonstrated antioxidant properties by reducing oxidative stress markers and increasing CAT activity [293,294]. Similar results have been reported in small groups of hypertensive patients [295,296]. Furthermore, the antioxidant properties of olmesartan and telmisartan have been observed in patients treated with these drugs [297,298].

Another example of a drug used to alleviate the symptoms of CAD is trimetazidine, which reduces oxidative stress markers such as MDA and lipid hydroperoxides and increases endothelium-dependent relaxation [299]. These effects were also observed in a prospective study involving patients with NSTEMI [300].

In addition to the above, Proprotein Convertase Subtilisin/Kexin 9 (PCSK9) inhibitors are a new therapeutic option in patients with hyperlipidemia. In clinical trials on large populations, they have been shown to be effective in lowering LDL cholesterol levels and reducing in endpoints [301]. Shortly after the introduction of this group of drugs, their beneficial effect on ox-LDL levels in blood plasma was reported [302]. Quite recently, PCSK9 inhibition was reported to suppress platelet activation and in vivo thrombosis by regulating oxidative stress [303]. Moreover, patients with acute coronary syndrome treated with a PCSK9 inhibitor displayed reduced ox-LDL levels, a key indicator of oxidative stress. In addition, PCSK-9 inhibitors have been reported to protect against myocardial ischemia–reperfusion injury [304]. Given these findings, it is reasonable to conclude that PCSK9 inhibitors might provide a therapeutic strategy for reducing cardiovascular risk related to oxidative stress.

Despite the information mentioned about drugs with antioxidant properties used in the treatment of atherosclerosis and coronary heart disease, the impact of specific antioxidants, such as coenzyme Q10 (CoQ10), flavonoids, carotenoids, and resveratrol in reducing morbidity and progression of the disease has also been extensively studied [305]. Results of several studies have shown that CoQ10 supplementation in patients with CAD significantly lowers total cholesterol and triglyceride levels while simultaneously increasing HDL-C concentrations [306]. Polyphenols, a diverse group of plant-derived antioxidants, have been widely studied for their cardiovascular protective effects [307]. The effects are attributed to their direct antioxidant properties, which include scavenging ROS and reactive nitrogen species. Resveratrol has also been shown to improve both the systolic and diastolic function of the left ventricle as well as the left ventricular ejection fraction in patients with CHD [308]. However, higher serum levels of carotenoids were associated with a reduced risk of elevated serum NT-proBNP levels, suggesting that carotenoids may help prevent cardiac overload [309,310].

Research has consistently demonstrated that antioxidants can help alleviate the toxic effects of drugs and chemicals, particularly by reducing oxidative damage in vital organs such as the liver, heart, and kidneys. Antioxidants, whether naturally occurring in fruits, vegetables, and beverages, or synthesized in laboratories, play a crucial role in counteracting oxidative stress caused by toxic exposures. For instance, antioxidants like vitamins C and E have shown promise in mitigating oxidative damage induced by nanoparticles, drugs, and environmental toxins [311]. By enhancing the body’s natural defense mechanisms, antioxidants offer a promising strategy for reducing drug-induced toxicity in cardiovascular and neurodegenerative conditions, as well as in cancer therapies. Recent studies have also highlighted their ability to alleviate toxicity in various organ systems, including the liver, heart, and nervous system, by modulating key biochemical pathways such as ROS scavenging and apoptosis regulation [312]. Furthermore, plant-derived antioxidants have been explored for their potential as adjuvant treatments, providing additional therapeutic benefits when combined with conventional drugs. These findings underscore the biomedical value of antioxidants in mitigating drug and chemical toxicity, improving patient outcomes, and reducing adverse side effects associated with pharmacological treatments. Together, these insights open avenues for novel therapeutic strategies to prevent or reduce the harmful effects of chemical and drug-induced toxicities.

Considering the above issues related to oxidative stress in cardiovascular diseases, it can be concluded that understanding the causes and effects of oxidative stress in these conditions will support the informed use of antioxidant drugs in patients, potentially accelerating improvements in their health.

## 5. Conclusions

This review provides a comprehensive summary of the current understanding of oxidative stress in the context of atherosclerosis and its contribution to the development and progression of cardiovascular diseases. Over recent decades, research has significantly advanced in identifying the role of oxidative stress as a key factor in atherosclerotic plaque formation, progression, and complications. Specifically, oxidative stress emerges early in atherosclerosis, amplifying inflammatory responses, contributing to endothelial dysfunction, and promoting plaque instability, which ultimately affects disease progression and therapy outcomes.

In reviewing the literature, several emerging trends have been identified that could reshape the landscape of atherosclerosis research and treatment. First, mitochondrial dysfunction, particularly the discovery of the mitochondrial permeability transition pore (mPTP), has become a focal point for new therapeutic strategies aimed at mitigating reperfusion injury. Second, the growing recognition of the diverse sources of ROS and their interrelationships suggests that targeting specific ROS production may offer more effective treatment approaches compared to broad-spectrum antioxidants. However, the clinical application of selective ROS inhibitors remains a challenge, as clinically viable compounds are yet to be developed. Lastly, the potential of oxidative stress products, such as oxLDL, MDA, IsoP, and 8-OHdG, as biomarkers for early-stage atherosclerosis and its complications, is gaining attention. These molecules show promise in enhancing diagnostic accuracy, monitoring disease progression, and assessing therapeutic outcomes, although further clinical validation is needed.

By synthesizing current knowledge and identifying key research directions, this review highlights the potential for targeted interventions in oxidative stress pathways, the need for more selective antioxidants, and the development of novel biomarkers that could improve the diagnosis and management of atherosclerosis. These emerging trends signal a shift toward more precise and personalized approaches in combating cardiovascular diseases associated with atherosclerosis.

## Figures and Tables

**Table 1:** The most common clinical presentations as a consequence of atherosclerosis.

Clinical Presentation	Location	Consequences/ Complications
CAD	Coronary arteries	Myocardial infarction
Carotid atherosclerosis	Carotid arteries	TIA/Stroke
Chronic limb ischemia	Peripheral arterial disease	Acute limb ischemia, CLTI
Intestinal ischemia	Mesenteric arteries	Acute intestinal ischemia
Renal artery atherosclerosis	Renal arteries	Hypertension, pulmonary oedema, renal atrophy

**Table 2:** Comparison of the properties of normal and activated endothelium.

Parameter	Normal Endothelium	Activated Endothelium
Tightness	preserved continuity of endothelial cells, glycocalyx	barrier unsealing, glycocalyx damage
Coagulation	inhibition of coagulation: NO, prostacyclin, glycocalyx, thrombomodulin, tissue factor inhibitor, activation of fibrinolysis	activation of coagulation: decrease in NO and prostacyclin production, von Willebrand factor, P-selactin, plasminogen inhibitor
Inflammation	anti-inflammatory and anti-adhesive properties	pro-inflammatory properties: expression of adhesion molecules, cytokines and chemokines

**Table 3:** ROS-related factors as a trigger of plaque instability and rapture.

ROS Action	Mechanism Which Leads to Plaque Rapture
Oxidized lipids	Recruitment of inflammatory cells and their activation—ruptured plaques contain more inflammatory cells [32]
Oxidized LDL	Foam cells formation and apoptosis, lipid core enlargement [33]
	Thrombus formation [34]
	Cyclooxygenase 2/membrane-bound prostaglandin E synthase 1 upregulation and inflammatory response [35]
	Endothelial cells apoptosis [36]
Activation of matrix metalloproteinases by ROS	Digestion of collagen fibers, thinning, fibrous cap [37]
	Easier monocyte/macrophage accumulation into plaques [38]
	Plaque neovascularization (and as a result intraplaque hemorrhages) by mobilizing VEGF and degradation of extracellular matrix fibers [39]
	VSMCs apoptosis as a result of N-cadherin degradation [40]
Endothelial dysfunction, NO degradation by ROS	Vasospasm [41]
	Loss of the direct anti-inflammatory effect of NO [42]

**Table 4:** Changes in the activity of selected antioxidant enzymes and the level of oxidative markers of lipid, protein, and nucleic acid modifications in atherosclerosis and ACS compared to the healthy controls.

Analyzed Parameters	Atherosclerosis	Reference	ACS	Reference
SOD	↓ (↑)	[125,128,129]	↓ ↓ (↑)	[138,249]
CAT	↓	[128,131]	↓ ↓ (↑)	[138,249]
GPx	↓ (↔)	[126,132]	↓ ↓ (↑)	[141,142,143]
TAS	↓	[125]	↓ ↓	[133]
oxLDL	↑	[169,250]	↑ ↑ (↔)	[172,177,179]
MDA	↑	[153,251]	↑ ↑	[181,249]
HNE	↑	[154,252]	↑ ↑	[182,183]
IsoP	↑	[161,253,254]	↑ ↑	[186,187]
S-glutathionylation	↑	[193]	↑ (↑ ↑)	[216,217,255]
Nitrotyrosine	↑	[201,256]	↔	[221,230]
IMA	↑	[211,214]	↑ ↑	[229,233]
Protein carbonyl	↑	[206,208]	↑	[222,257]
Modified guanine	↑	[235,241]	↑ ↑	[181,243,245]

↑ activity/level increase; ↓ activity/level increase; ↔ no change in activity/level; the arrow in brackets indicates a less frequently observed or questionable effect; IMA—ischemia-modified albumin; TAS—total antioxidant status.

## Data Availability

The data are available in this article.

## References

[1] Björkegren J.L.M., Lusis A.J. (2022). Atherosclerosis: Recent Developments. Cell.

[2] (2024). WHO. https://www.who.int/data/gho/data/themes/mortality-and-global-health-estimates/global-health-estimates-leading-causes-of-dalys.

[3] Pahwa R., Jialal I. (2024). Atherosclerosis. StatPearls.

[4] Heart Statistics. https://www.bhf.org.uk/what-we-do/our-research/heart-statistics.

[5] Timmis A., Vardas P., Townsend N., Torbica A., Katus H., De Smedt D., Gale C.P., Maggioni A.P., Petersen S.E., Huculeci R. (2022). European Society of Cardiology: Cardiovascular Disease Statistics 2021. Eur. Heart J..

[6] Knuuti J., Wijns W., Saraste A., Capodanno D., Barbato E., Funck-Brentano C., Prescott E., Storey R.F., Deaton C., Cuisset T. (2020). 2019 ESC Guidelines for the Diagnosis and Management of Chronic Coronary Syndromes. Eur. Heart J..

[7] Thygesen K., Alpert J.S., Jaffe A.S., Chaitman B.R., Bax J.J., Morrow D.A., White H.D., The Executive Group on behalf of the Joint European Society of Cardiology (ESC)/American College of Cardiology (ACC)/American Heart Association (AHA)/World Heart Federation (WHF) Task Force for the Universal Definition of Myocardial Infarction (2018). Fourth Universal Definition of Myocardial Infarction (2018). Circulation.

[8] Byrne R.A., Rossello X., Coughlan J.J., Barbato E., Berry C., Chieffo A., Claeys M.J., Dan G.-A., Dweck M.R., Galbraith M. (2024). 2023 ESC Guidelines for the Management of Acute Coronary Syndromes: Developed by the Task Force on the Management of Acute Coronary Syndromes of the European Society of Cardiology (ESC). Eur. Heart J. Acute Cardiovasc. Care.

[9] Zaki H.A., Shaban A.E., Shaban A.E., Shaban E.E. (2022). Interpretation of Cardiac and Non-Cardiac Causes of Elevated Troponin T Levels in Non-Acute Coronary Syndrome Patients in the Emergency Department. Cureus.

[10] Zhang H., Hu H., Zhai C., Jing L., Tian H. (2024). Cardioprotective Strategies After Ischemia-Reperfusion Injury. Am. J. Cardiovasc. Drugs.

[11] Kattoor A.J., Pothineni N.V.K., Palagiri D., Mehta J.L. (2017). Oxidative Stress in Atherosclerosis. Curr. Atheroscler. Rep..

[12] Arenas de Larriva A.P., Limia-Pérez L., Alcalá-Díaz J.F., Alonso A., López-Miranda J., Delgado-Lista J. (2020). Ceruloplasmin and Coronary Heart Disease-A Systematic Review. Nutrients.

[13] Korczowska-Łącka I., Słowikowski B., Piekut T., Hurła M., Banaszek N., Szymanowicz O., Jagodziński P.P., Kozubski W., Permoda-Pachuta A., Dorszewska J. (2023). Disorders of Endogenous and Exogenous Antioxidants in Neurological Diseases. Antioxidants.

[14] Griendling K.K., Camargo L.L., Rios F., Alves-Lopes R., Montezano A.C., Touyz R.M. (2021). Oxidative Stress and Hypertension. Circ. Res..

[15] Yuan T., Yang T., Chen H., Fu D., Hu Y., Wang J., Yuan Q., Yu H., Xu W., Xie X. (2019). New Insights into Oxidative Stress and Inflammation during Diabetes Mellitus-Accelerated Atherosclerosis. Redox Biol..

[16] Świątkiewicz I., Wróblewski M., Nuszkiewicz J., Sutkowy P., Wróblewska J., Woźniak A. (2023). The Role of Oxidative Stress Enhanced by Adiposity in Cardiometabolic Diseases. Int. J. Mol. Sci..

[17] Anrather J., Racchumi G., Iadecola C. (2006). NF-kappaB Regulates Phagocytic NADPH Oxidase by Inducing the Expression of Gp91phox. J. Biol. Chem..

[18] Kamceva G., Arsova-Sarafinovska Z., Ruskovska T., Zdravkovska M., Kamceva-Panova L., Stikova E. (2016). Cigarette Smoking and Oxidative Stress in Patients with Coronary Artery Disease. Open Access Maced. J. Med. Sci..

[19] Ganjali S., Keshavarz R., Hosseini S., Mansouri A., Mannarino M.R., Pirro M., Jamialahmadi T., Sahebkar A. (2021). Evaluation of Oxidative Stress Status in Familial Hypercholesterolemia. J. Clin. Med..

[20] Hejazi K., Ghahremani Moghaddam M., Darzabi T. (2019). Effects of an 8-Week Aerobic Exercise Program on Some Indicators of Oxidative Stress in Elderly Women. Iran. J. Ageing.

[21] Powers S.K., Deminice R., Ozdemir M., Yoshihara T., Bomkamp M.P., Hyatt H. (2020). Exercise-Induced Oxidative Stress: Friend or Foe?. J. Sport. Health Sci..

[22] Bentzon J.F., Otsuka F., Virmani R., Falk E. (2014). Mechanisms of Plaque Formation and Rupture. Circ. Res..

[23] Alonso-Herranz L., Albarrán-Juárez J., Bentzon J.F. (2023). Mechanisms of Fibrous Cap Formation in Atherosclerosis. Front. Cardiovasc. Med..

[24] Zhang F., Wang C., Wang H., Lu M., Li Y., Feng H., Lin J., Yuan Z., Wang X. (2013). Ox-LDL Promotes Migration and Adhesion of Bone Marrow-Derived Mesenchymal Stem Cells via Regulation of MCP-1 Expression. Mediat. Inflamm..

[25] Wang B., Ge Z., Cheng Z., Zhao Z. (2017). Tanshinone IIA Suppresses the Progression of Atherosclerosis by Inhibiting the Apoptosis of Vascular Smooth Muscle Cells and the Proliferation and Migration of Macrophages Induced by Ox-LDL. Biol. Open.

[26] Liu J., Ren Y., Kang L., Zhang L. (2014). Oxidized Low-Density Lipoprotein Increases the Proliferation and Migration of Human Coronary Artery Smooth Muscle Cells through the Upregulation of Osteopontin. Int. J. Mol. Med..

[27] Liao Y., Zhu E., Zhou W. (2021). Ox-LDL Aggravates the Oxidative Stress and Inflammatory Responses of THP-1 Macrophages by Reducing the Inhibition Effect of miR-491-5p on MMP-9. Front. Cardiovasc. Med..

[28] Kim M., Yoo H.J., Lee D., Lee J.H. (2020). Oxidized LDL Induces Procoagulant Profiles by Increasing Lysophosphatidylcholine Levels, Lysophosphatidylethanolamine Levels, and Lp-PLA2 Activity in Borderline Hypercholesterolemia. Nutr. Metab. Cardiovasc. Dis..

[29] Van der Veken B., De Meyer G.R.Y., Martinet W. (2018). Axitinib Attenuates Intraplaque Angiogenesis, Haemorrhages and Plaque Destabilization in Mice. Vasc. Pharmacol..

[30] Shioi A., Ikari Y. (2018). Plaque Calcification During Atherosclerosis Progression and Regression. J. Atheroscler. Thromb..

[31] Salekeen R., Haider A.N., Akhter F., Billah M.M., Islam M.E., Didarul Islam K.M. (2022). Lipid Oxidation in Pathophysiology of Atherosclerosis: Current Understanding and Therapeutic Strategies. Int. J. Cardiol. Cardiovasc. Risk Prev..

[32] Mehu M., Narasimhulu C.A., Singla D.K. (2022). Inflammatory Cells in Atherosclerosis. Antioxidants.

[33] Moore K.J., Tabas I. (2011). Macrophages in the Pathogenesis of Atherosclerosis. Cell.

[34] Obermayer G., Afonyushkin T., Binder C.J. (2018). Oxidized Low-Density Lipoprotein in Inflammation-Driven Thrombosis. J. Thromb. Haemost..

[35] Gargiulo S., Rossin D., Testa G., Gamba P., Staurenghi E., Biasi F., Poli G., Leonarduzzi G. (2018). Up-Regulation of COX-2 and mPGES-1 by 27-Hydroxycholesterol and 4-Hydroxynonenal: A Crucial Role in Atherosclerotic Plaque Instability. Free Radic. Biol. Med..

[36] Chen J., Mehta J.L., Haider N., Zhang X., Narula J., Li D. (2004). Role of Caspases in Ox-LDL-Induced Apoptotic Cascade in Human Coronary Artery Endothelial Cells. Circ. Res..

[37] O’Toole T.E., Zheng Y.-T., Hellmann J., Conklin D.J., Barski O., Bhatnagar A. (2009). Acrolein Activates Matrix Metalloproteinases by Increasing Reactive Oxygen Species in Macrophages. Toxicol. Appl. Pharmacol..

[38] Di Gregoli K., George S.J., Jackson C.L., Newby A.C., Johnson J.L. (2016). Differential Effects of Tissue Inhibitor of Metalloproteinase (TIMP)-1 and TIMP-2 on Atherosclerosis and Monocyte/Macrophage Invasion. Cardiovasc. Res..

[39] Butoi E., Gan A.M., Tucureanu M.M., Stan D., Macarie R.D., Constantinescu C., Calin M., Simionescu M., Manduteanu I. (2016). Cross-Talk between Macrophages and Smooth Muscle Cells Impairs Collagen and Metalloprotease Synthesis and Promotes Angiogenesis. Biochim. Biophys. Acta.

[40] Williams H., Johnson J.L., Jackson C.L., White S.J., George S.J. (2010). MMP-7 Mediates Cleavage of N-Cadherin and Promotes Smooth Muscle Cell Apoptosis. Cardiovasc. Res..

[41] Chen J.-Y., Ye Z.-X., Wang X.-F., Chang J., Yang M.-W., Zhong H.-H., Hong F.-F., Yang S.-L. (2018). Nitric Oxide Bioavailability Dysfunction Involves in Atherosclerosis. Biomed. Pharmacother..

[42] Ning K., Wang M.-J., Lin G., Zhang Y.-L., Li M.-Y., Yang B.-F., Chen Y., Huang Y., Li Z.-M., Huang Y.-J. (2020). eNOS-Nitric Oxide System Contributes to a Novel Antiatherogenic Effect of Leonurine via Inflammation Inhibition and Plaque Stabilization. J. Pharmacol. Exp. Ther..

[43] Chen Q., Wang Q., Zhu J., Xiao Q., Zhang L. (2018). Reactive Oxygen Species: Key Regulators in Vascular Health and Diseases. Br. J. Pharmacol..

[44] Vermot A., Petit-Härtlein I., Smith S.M.E., Fieschi F. (2021). NADPH Oxidases (NOX): An Overview from Discovery, Molecular Mechanisms to Physiology and Pathology. Antioxidants.

[45] Burtenshaw D., Kitching M., Redmond E.M., Megson I.L., Cahill P.A. (2019). Reactive Oxygen Species (ROS), Intimal Thickening, and Subclinical Atherosclerotic Disease. Front. Cardiovasc. Med..

[46] Lapouge K., Smith S.J.M., Groemping Y., Rittinger K. (2002). Architecture of the P40-P47-P67phox Complex in the Resting State of the NADPH Oxidase. A Central Role for P67phox. J. Biol. Chem..

[47] Konior A., Schramm A., Czesnikiewicz-Guzik M., Guzik T.J. (2014). NADPH Oxidases in Vascular Pathology. Antioxid. Redox Signal..

[48] Gray S.P., Di Marco E., Okabe J., Szyndralewiez C., Heitz F., Montezano A.C., de Haan J.B., Koulis C., El-Osta A., Andrews K.L. (2013). NADPH Oxidase 1 Plays a Key Role in Diabetes Mellitus–Accelerated Atherosclerosis. Circulation.

[49] Lee M.Y., San Martin A., Mehta P.K., Dikalova A.E., Garrido A.M., Datla S.R., Lyons E., Krause K.-H., Banfi B., Lambeth J.D. (2009). Mechanisms of Vascular Smooth Muscle NADPH Oxidase 1 (Nox1) Contribution to Injury-Induced Neointimal Formation. Arterioscler. Thromb. Vasc. Biol..

[50] Vendrov A.E., Sumida A., Canugovi C., Lozhkin A., Hayami T., Madamanchi N.R., Runge M.S. (2019). NOXA1-Dependent NADPH Oxidase Regulates Redox Signaling and Phenotype of Vascular Smooth Muscle Cell during Atherogenesis. Redox Biol..

[51] Judkins C.P., Diep H., Broughton B.R.S., Mast A.E., Hooker E.U., Miller A.A., Selemidis S., Dusting G.J., Sobey C.G., Drummond G.R. (2010). Direct Evidence of a Role for Nox2 in Superoxide Production, Reduced Nitric Oxide Bioavailability, and Early Atherosclerotic Plaque Formation in ApoE^−/−^ Mice. Am. J. Physiol. Heart Circ. Physiol..

[52] Quesada I.M., Lucero A., Amaya C., Meijles D.N., Cifuentes M.E., Pagano P.J., Castro C. (2015). Selective Inactivation of NADPH Oxidase 2 Causes Regression of Vascularization and the Size and Stability of Atherosclerotic Plaques. Atherosclerosis.

[53] da Silva J.F., Alves J.V., Silva-Neto J.A., Costa R.M., Neves K.B., Alves-Lopes R., Carmargo L.L., Rios F.J., Montezano A.C., Touyz R.M. (2021). Lysophosphatidylcholine Induces Oxidative Stress in Human Endothelial Cells via NOX5 Activation—Implications in Atherosclerosis. Clin. Sci..

[54] Di Marco E., Gray S.P., Kennedy K., Szyndralewiez C., Lyle A.N., Lassègue B., Griendling K.K., Cooper M.E., Schmidt H.H.H.W., Jandeleit-Dahm K.A.M. (2016). NOX4-Derived Reactive Oxygen Species Limit Fibrosis and Inhibit Proliferation of Vascular Smooth Muscle Cells in Diabetic Atherosclerosis. Free Radic. Biol. Med..

[55] Yu W., Li S., Wu H., Hu P., Chen L., Zeng C., Tong X. (2021). Endothelial Nox4 Dysfunction Aggravates Atherosclerosis by Inducing Endoplasmic Reticulum Stress and Soluble Epoxide Hydrolase. Free Radic. Biol. Med..

[56] Tong X., Khandelwal A.R., Wu X., Xu Z., Yu W., Chen C., Zhao W., Yang J., Qin Z., Weisbrod R.M. (2016). Pro-Atherogenic Role of Smooth Muscle Nox4-Based NADPH Oxidase. J. Mol. Cell Cardiol..

[57] Diebold I., Petry A., Hess J., Görlach A. (2010). The NADPH Oxidase Subunit NOX4 Is a New Target Gene of the Hypoxia-Inducible Factor-1. Mol. Biol. Cell.

[58] Goyal P., Weissmann N., Grimminger F., Hegel C., Bader L., Rose F., Fink L., Ghofrani H.A., Schermuly R.T., Schmidt H.H.H.W. (2004). Upregulation of NAD(P)H Oxidase 1 in Hypoxia Activates Hypoxia-Inducible Factor 1 via Increase in Reactive Oxygen Species. Free Radic. Biol. Med..

[59] Zhao W., Zhao D., Yan R., Sun Y. (2009). Cardiac Oxidative Stress and Remodeling Following Infarction: Role of NADPH Oxidase. Cardiovasc. Pathol..

[60] Kleinschnitz C., Grund H., Wingler K., Armitage M.E., Jones E., Mittal M., Barit D., Schwarz T., Geis C., Kraft P. (2010). Post-Stroke Inhibition of Induced NADPH Oxidase Type 4 Prevents Oxidative Stress and Neurodegeneration. PLoS Biol..

[61] Braunersreuther V., Montecucco F., Asrih M., Pelli G., Galan K., Frias M., Burger F., Quinderé A.L.G., Montessuit C., Krause K.-H. (2013). Role of NADPH Oxidase Isoforms NOX1, NOX2 and NOX4 in Myocardial Ischemia/Reperfusion Injury. J. Mol. Cell Cardiol..

[62] Sirker A., Murdoch C.E., Protti A., Sawyer G.J., Santos C.X.C., Martin D., Zhang X., Brewer A.C., Zhang M., Shah A.M. (2016). Cell-Specific Effects of Nox2 on the Acute and Chronic Response to Myocardial Infarction. J. Mol. Cell Cardiol..

[63] Nolfi-Donegan D., Braganza A., Shiva S. (2020). Mitochondrial Electron Transport Chain: Oxidative Phosphorylation, Oxidant Production, and Methods of Measurement. Redox Biol..

[64] Andrés C.M.C., Pérez de la Lastra J.M., Andrés Juan C., Plou F.J., Pérez-Lebeña E. (2023). Superoxide Anion Chemistry—Its Role at the Core of the Innate Immunity. Int. J. Mol. Sci..

[65] Mazat J.-P., Devin A., Ransac S. (2020). Modelling Mitochondrial ROS Production by the Respiratory Chain. Cell. Mol. Life Sci..

[66] Aon M.A., Cortassa S., O’Rourke B. (2010). Redox-Optimized ROS Balance: A Unifying Hypothesis. Biochim. Biophys. Acta.

[67] Kotova E.A., Antonenko Y.N. (2022). Fifty Years of Research on Protonophores: Mitochondrial Uncoupling As a Basis for Therapeutic Action. Acta Naturae.

[68] Ježek P., Holendová B., Garlid K.D., Jabůrek M. (2018). Mitochondrial Uncoupling Proteins: Subtle Regulators of Cellular Redox SignalingReviewing Editors: Jerzy Beltowski, Joseph Burgoyne, Gabor Csanyi, Sergey Dikalov, Frank Krause, Anibal Vercesi, and Jeremy Ward. Antioxid. Redox Signal..

[69] Heinen A., Camara A.K.S., Aldakkak M., Rhodes S.S., Riess M.L., Stowe D.F. (2007). Mitochondrial Ca^2+^-Induced K^+^ Influx Increases Respiration and Enhances ROS Production While Maintaining Membrane Potential. Am. J. Physiol. Cell Physiol..

[70] Andrukhiv A., Costa A.D., West I.C., Garlid K.D. (2006). Opening mitoKATP Increases Superoxide Generation from Complex I of the Electron Transport Chain. Am. J. Physiol. Heart Circ. Physiol..

[71] Behera R., Sharma V., Grewal A.K., Kumar A., Arora B., Najda A., Albadrani G.M., Altyar A.E., Abdel-Daim M.M., Singh T.G. (2023). Mechanistic Correlation between Mitochondrial Permeability Transition Pores and Mitochondrial ATP Dependent Potassium Channels in Ischemia Reperfusion. Biomed. Pharmacother..

[72] Pryde K.R., Hirst J. (2011). Superoxide Is Produced by the Reduced Flavin in Mitochondrial Complex I: A Single, Unified Mechanism That Applies during Both Forward and Reverse Electron Transfer. J. Biol. Chem..

[73] Murphy M.P. (2009). How Mitochondria Produce Reactive Oxygen Species. Biochem. J..

[74] Chouchani E.T., Pell V.R., James A.M., Work L.M., Saeb-Parsy K., Frezza C., Krieg T., Murphy M.P. (2016). A Unifying Mechanism for Mitochondrial Superoxide Production during Ischemia-Reperfusion Injury. Cell Metab..

[75] Manni M.E., Rigacci S., Borchi E., Bargelli V., Miceli C., Giordano C., Raimondi L., Nediani C. (2016). Monoamine Oxidase Is Overactivated in Left and Right Ventricles from Ischemic Hearts: An Intriguing Therapeutic Target. Oxid. Med. Cell. Longev..

[76] Shi Y., Hou S.-A. (2021). Protective Effects of Metformin against Myocardial Ischemia-reperfusion Injury via AMPK-dependent Suppression of NOX4. Mol. Med. Rep..

[77] Giorgio M., Migliaccio E., Orsini F., Paolucci D., Moroni M., Contursi C., Pelliccia G., Luzi L., Minucci S., Marcaccio M. (2005). Electron Transfer between Cytochrome c and p66Shc Generates Reactive Oxygen Species That Trigger Mitochondrial Apoptosis. Cell.

[78] Zorov D.B., Isaev N.K., Plotnikov E.Y., Silachev D.N., Zorova L.D., Pevzner I.B., Morosanova M.A., Jankauskas S.S., Zorov S.D., Babenko V.A. (2013). Perspectives of Mitochondrial Medicine. Biochemistry.

[79] Boyenle I.D., Oyedele A.K., Ogunlana A.T., Adeyemo A.F., Oyelere F.S., Akinola O.B., Adelusi T.I., Ehigie L.O., Ehigie A.F. (2022). Targeting the Mitochondrial Permeability Transition Pore for Drug Discovery: Challenges and Opportunities. Mitochondrion.

[80] Seidlmayer L.K., Juettner V.V., Kettlewell S., Pavlov E.V., Blatter L.A., Dedkova E.N. (2015). Distinct mPTP Activation Mechanisms in Ischaemia-Reperfusion: Contributions of Ca^2+^, ROS, pH, and Inorganic Polyphosphate. Cardiovasc. Res..

[81] Yalamanchili K., Afzal N., Boyman L., Mannella C.A., Lederer W.J., Jafri M.S. (2022). Understanding the Dynamics of the Transient and Permanent Opening Events of the Mitochondrial Permeability Transition Pore with a Novel Stochastic Model. Membranes.

[82] Chapa-Dubocq X.R., Rodríguez-Graciani K.M., Escobales N., Javadov S. (2023). Mitochondrial Volume Regulation and Swelling Mechanisms in Cardiomyocytes. Antioxidants.

[83] Gómez-Crisóstomo N.P., López-Marure R., Zapata E., Zazueta C., Martínez-Abundis E. (2013). Bax Induces Cytochrome c Release by Multiple Mechanisms in Mitochondria from MCF7 Cells. J. Bioenerg. Biomembr..

[84] Mendoza A., Patel P., Robichaux D., Ramirez D., Karch J. (2024). Inhibition of the mPTP and Lipid Peroxidation Is Additively Protective Against I/R Injury. Circ. Res..

[85] Bulua A.C., Simon A., Maddipati R., Pelletier M., Park H., Kim K.-Y., Sack M.N., Kastner D.L., Siegel R.M. (2011). Mitochondrial Reactive Oxygen Species Promote Production of Proinflammatory Cytokines and Are Elevated in TNFR1-Associated Periodic Syndrome (TRAPS). J. Exp. Med..

[86] Li X., Fang P., Li Y., Kuo Y.-M., Andrews A.J., Nanayakkara G., Johnson C., Fu H., Shan H., Du F. (2016). Mitochondrial Reactive Oxygen Species Mediate Lysophosphatidylcholine-Induced Endothelial Cell Activation. Arterioscler. Thromb. Vasc. Biol..

[87] Yu E.P., Reinhold J., Yu H., Starks L., Uryga A.K., Foote K., Finigan A., Figg N., Pung Y.-F., Logan A. (2017). Mitochondrial Respiration Is Reduced in Atherosclerosis, Promoting Necrotic Core Formation and Reducing Relative Fibrous Cap Thickness. Arterioscler. Thromb. Vasc. Biol..

[88] Yu E., Calvert P.A., Mercer J.R., Harrison J., Baker L., Figg N.L., Kumar S., Wang J.C., Hurst L.A., Obaid D.R. (2013). Mitochondrial DNA Damage Can Promote Atherosclerosis Independently of Reactive Oxygen Species through Effects on Smooth Muscle Cells and Monocytes and Correlates with Higher-Risk Plaques in Humans. Circulation.

[89] Sudakov N.P., Apartsin K.A., Lepekhova S.A., Nikiforov S.B., Katyshev A.I., Lifshits G.I., Vybivantseva A.V., Konstantinov Y.M. (2017). The Level of Free Circulating Mitochondrial DNA in Blood as Predictor of Death in Case of Acute Coronary Syndrome. Eur. J. Med. Res..

[90] Vendrov A.E., Vendrov K.C., Smith A., Yuan J., Sumida A., Robidoux J., Runge M.S., Madamanchi N.R. (2015). NOX4 NADPH Oxidase-Dependent Mitochondrial Oxidative Stress in Aging-Associated Cardiovascular Disease. Antioxid. Redox Signal..

[91] Wang Y., Wang W., Wang N., Tall A.R., Tabas I. (2017). Mitochondrial Oxidative Stress Promotes Atherosclerosis and Neutrophil Extracellular Traps in Aged Mice. Arterioscler. Thromb. Vasc. Biol..

[92] Wang Y., Wang G.Z., Rabinovitch P.S., Tabas I. (2014). Macrophage Mitochondrial Oxidative Stress Promotes Atherosclerosis and Nuclear Factor-κB-Mediated Inflammation in Macrophages. Circ. Res..

[93] Montezano A.C., Touyz R.M. (2012). Reactive Oxygen Species and Endothelial Function—Role of Nitric Oxide Synthase Uncoupling and Nox Family Nicotinamide Adenine Dinucleotide Phosphate Oxidases. Basic. Clin. Pharmacol. Toxicol..

[94] Channon K.M. (2021). Tetrahydrobiopterin and Nitric Oxide Synthase Recouplers. Handb. Exp. Pharmacol..

[95] Wei S.-J., Cheng L., Liang E.-S., Wang Q., Zhou S.-N., Xu H., Hui L.-H., Ge Z.-M., Zhang M.-X. (2017). Poly(ADP-Ribose) Polymerase 1 Deficiency Increases Nitric Oxide Production and Attenuates Aortic Atherogenesis through Downregulation of Arginase II. Clin. Exp. Pharmacol. Physiol..

[96] Barros C.D.S., Livramento J.B., Mouro M.G., Higa E.M.S., Moraes C.T., Tengan C.H. (2021). L-Arginine Reduces Nitro-Oxidative Stress in Cultured Cells with Mitochondrial Deficiency. Nutrients.

[97] Chen C.-A., Wang T.-Y., Varadharaj S., Reyes L.A., Hemann C., Talukder M.A.H., Chen Y.-R., Druhan L.J., Zweier J.L. (2010). S-Glutathionylation Uncouples eNOS and Regulates Its Cellular and Vascular Function. Nature.

[98] Förstermann U., Xia N., Li H. (2017). Roles of Vascular Oxidative Stress and Nitric Oxide in the Pathogenesis of Atherosclerosis. Circ. Res..

[99] Oemar B.S., Tschudi M.R., Godoy N., Brovkovich V., Malinski T., Lüscher T.F. (1998). Reduced Endothelial Nitric Oxide Synthase Expression and Production in Human Atherosclerosis. Circulation.

[100] Mahdi A., Kövamees O., Pernow J. (2020). Improvement in Endothelial Function in Cardiovascular Disease—Is Arginase the Target?. Int. J. Cardiol..

[101] Li L., Chen W., Rezvan A., Jo H., Harrison D.G. (2011). Tetrahydrobiopterin Deficiency and Nitric Oxide Synthase Uncoupling Contribute to Atherosclerosis Induced by Disturbed Flow. Arterioscler. Thromb. Vasc. Biol..

[102] Hu L., Wang J., Zhu H., Wu X., Zhou L., Song Y., Zhu S., Hao M., Liu C., Fan Y. (2016). Ischemic Postconditioning Protects the Heart against Ischemia-Reperfusion Injury via Neuronal Nitric Oxide Synthase in the Sarcoplasmic Reticulum and Mitochondria. Cell Death Dis..

[103] Suno K., Shingu Y., Wakasa S. (2022). Protective Effects of Trehalose Preconditioning on Cardiac and Coronary Endothelial Function through eNOS Signaling Pathway in a Rat Model of Ischemia-Reperfusion Injury. Mol. Cell. Biochem..

[104] Kuhlencordt P.J., Chen J., Han F., Astern J., Huang P.L. (2001). Genetic Deficiency of Inducible Nitric Oxide Synthase Reduces Atherosclerosis and Lowers Plasma Lipid Peroxides in Apolipoprotein E-Knockout Mice. Circulation.

[105] Depre C., Havaux X., Renkin J., Vanoverschelde J.L., Wijns W. (1999). Expression of Inducible Nitric Oxide Synthase in Human Coronary Atherosclerotic Plaque. Cardiovasc. Res..

[106] Wilmes V., Scheiper S., Roehr W., Niess C., Kippenberger S., Steinhorst K., Verhoff M.A., Kauferstein S. (2020). Increased Inducible Nitric Oxide Synthase (iNOS) Expression in Human Myocardial Infarction. Int. J. Leg. Med..

[107] Jeddi S., Ghasemi A., Asgari A., Nezami-Asl A. (2018). Role of Inducible Nitric Oxide Synthase in Myocardial Ischemia-Reperfusion Injury in Sleep-Deprived Rats. Sleep Breath..

[108] Antoniades C., Shirodaria C., Leeson P., Antonopoulos A., Warrick N., Van-Assche T., Cunnington C., Tousoulis D., Pillai R., Ratnatunga C. (2009). Association of Plasma Asymmetrical Dimethylarginine (ADMA) with Elevated Vascular Superoxide Production and Endothelial Nitric Oxide Synthase Uncoupling: Implications for Endothelial Function in Human Atherosclerosis. Eur. Heart J..

[109] Yu W., Cheng J.-D. (2020). Uric Acid and Cardiovascular Disease: An Update From Molecular Mechanism to Clinical Perspective. Front. Pharmacol..

[110] Dai X.-M., Wei L., Ma L.-L., Chen H.-Y., Zhang Z.-J., Ji Z.-F., Wu W.-L., Ma L.-Y., Kong X.-F., Jiang L.-D. (2015). Serum Uric Acid and Its Relationship with Cardiovascular Risk Profile in Chinese Patients with Early-Onset Coronary Artery Disease. Clin. Rheumatol..

[111] Boban M., Kocic G., Radenkovic S., Pavlovic R., Cvetkovic T., Deljanin-Ilic M., Ilic S., Bobana M.D., Djindjic B., Stojanovic D. (2014). Circulating Purine Compounds, Uric Acid, and Xanthine Oxidase/Dehydrogenase Relationship in Essential Hypertension and End Stage Renal Disease. Ren. Fail..

[112] Gondouin B., Jourde-Chiche N., Sallee M., Dou L., Cerini C., Loundou A., Morange S., Berland Y., Burtey S., Brunet P. (2015). Plasma Xanthine Oxidase Activity Is Predictive of Cardiovascular Disease in Patients with Chronic Kidney Disease, Independently of Uric Acid Levels. Nephron.

[113] Klisic A., Kocic G., Kavaric N., Jovanovic M., Stanisic V., Ninic A. (2018). Xanthine Oxidase and Uric Acid as Independent Predictors of Albuminuria in Patients with Diabetes Mellitus Type 2. Clin. Exp. Med..

[114] Richette P., Poitou C., Manivet P., Denis J., Bouillot J.-L., Clément K., Oppert J.-M., Bardin T. (2016). Weight Loss, Xanthine Oxidase, and Serum Urate Levels: A Prospective Longitudinal Study of Obese Patients. Arthritis Care Res..

[115] Alrouji M., Manouchehrinia A., Aram J., Alotaibi A., Alhajlah S., Almuhanna Y., Alomeir O., Shamsi A., Gran B., Constantinescu C.S. (2023). Investigating the Effect of Cigarette Smoking on Serum Uric Acid Levels in Multiple Sclerosis Patients: A Cross Sectional Study. Brain Sci..

[116] Mori N., Saito Y., Saito K., Matsuoka T., Tateishi K., Kadohira T., Kitahara H., Fujimoto Y., Kobayashi Y. (2020). Relation of Plasma Xanthine Oxidoreductase Activity to Coronary Lipid Core Plaques Assessed by Near-Infrared Spectroscopy Intravascular Ultrasound in Patients With Stable Coronary Artery Disease. Am. J. Cardiol..

[117] Kushiyama A., Okubo H., Sakoda H., Kikuchi T., Fujishiro M., Sato H., Kushiyama S., Iwashita M., Nishimura F., Fukushima T. (2012). Xanthine Oxidoreductase Is Involved in Macrophage Foam Cell Formation and Atherosclerosis Development. Arterioscler. Thromb. Vasc. Biol..

[118] Nomura J., Busso N., Ives A., Matsui C., Tsujimoto S., Shirakura T., Tamura M., Kobayashi T., So A., Yamanaka Y. (2014). Xanthine Oxidase Inhibition by Febuxostat Attenuates Experimental Atherosclerosis in Mice. Sci. Rep..

[119] Ganji M., Nardi V., Prasad M., Jordan K.L., Bois M.C., Franchi F., Zhu X.Y., Tang H., Young M.D., Lerman L.O. (2021). Carotid Plaques From Symptomatic Patients Are Characterized by Local Increase in Xanthine Oxidase Expression. Stroke.

[120] Raghuvanshi R., Kaul A., Bhakuni P., Mishra A., Misra M.K. (2007). Xanthine Oxidase as a Marker of Myocardial Infarction. Indian J. Clin. Biochem..

[121] Rentoukas E., Tsarouhas K., Tsitsimpikou C., Lazaros G., Deftereos S., Vavetsi S. (2010). The Prognostic Impact of Allopurinol in Patients with Acute Myocardial Infarction Undergoing Primary Percutaneous Coronary Intervention. Int. J. Cardiol..

[122] Nambu H., Takada S., Maekawa S., Matsumoto J., Kakutani N., Furihata T., Shirakawa R., Katayama T., Nakajima T., Yamanashi K. (2021). Inhibition of Xanthine Oxidase in the Acute Phase of Myocardial Infarction Prevents Skeletal Muscle Abnormalities and Exercise Intolerance. Cardiovasc. Res..

[123] Bredemeier M., Lopes L.M., Eisenreich M.A., Hickmann S., Bongiorno G.K., d’Avila R., Morsch A.L.B., da Silva Stein F., Campos G.G.D. (2018). Xanthine Oxidase Inhibitors for Prevention of Cardiovascular Events: A Systematic Review and Meta-Analysis of Randomized Controlled Trials. BMC Cardiovasc. Disord..

[124] Gill D., Cameron A.C., Burgess S., Li X., Doherty D.J., Karhunen V., Abdul-Rahim A.H., Taylor-Rowan M., Zuber V., Tsao P.S. (2021). Urate, Blood Pressure, and Cardiovascular Disease: Evidence From Mendelian Randomization and Meta-Analysis of Clinical Trials. Hypertension.

[125] Ninić A., Bogavac-Stanojević N., Sopić M., Munjas J., Kotur-Stevuljević J., Miljković M., Gojković T., Kalimanovska-Oštrić D., Spasojević-Kalimanovska V. (2019). Superoxide Dismutase Isoenzymes Gene Expression in Peripheral Blood Mononuclear Cells in Patients with Coronary Artery Disease. J. Med. Biochem..

[126] Bastani A., Rajabi S., Daliran A., Saadat H., Karimi-Busheri F. (2018). Oxidant and Antioxidant Status in Coronary Artery Disease. Biomed. Rep..

[127] Barale C., Cavalot F., Frascaroli C., Bonomo K., Morotti A., Guerrasio A., Russo I. (2020). Association between High On-Aspirin Platelet Reactivity and Reduced Superoxide Dismutase Activity in Patients Affected by Type 2 Diabetes Mellitus or Primary Hypercholesterolemia. Int. J. Mol. Sci..

[128] Pytel E., Olszewska-Banaszczyk M., Koter-Michalak M., Broncel M. (2013). Increased Oxidative Stress and Decreased Membrane Fluidity in Erythrocytes of CAD Patients. Biochem. Cell Biol..

[129] Peng J.-R., Lu T.-T., Chang H.-T., Ge X., Huang B., Li W.-M. (2016). Elevated Levels of Plasma Superoxide Dismutases 1 and 2 in Patients with Coronary Artery Disease. Biomed. Res. Int..

[130] Zengin E., Sinning C., Zeller T., Rupprecht H.-J., Schnabel R.B., Lackner K.-J., Blankenberg S., Westermann D., Bickel C. (2015). Activity of Superoxide Dismutase Copper/Zinc Type and Prognosis in a Cohort of Patients with Coronary Artery Disease. Biomark. Med..

[131] Gupta S., Sodhi S., Mahajan V. (2009). Correlation of Antioxidants with Lipid Peroxidation and Lipid Profile in Patients Suffering from Coronary Artery Disease. Expert. Opin. Ther. Targets.

[132] Yang S., Jensen M.K., Rimm E.B., Willett W., Wu T. (2014). Erythrocyte Superoxide Dismutase, Glutathione Peroxidase, and Catalase Activities and Risk of Coronary Heart Disease in Generally Healthy Women: A Prospective Study. Am. J. Epidemiol..

[133] Guclu K., Celik M. (2020). Prognostic Value of Inflammation Parameters in Patients With Non-ST Elevation Acute Coronary Syndromes. Angiology.

[134] Aksoy S., Cam N., Gurkan U., Oz D., Özden K., Altay S., Durmus G., Agirbasli M. (2012). Oxidative Stress and Severity of Coronary Artery Disease in Young Smokers with Acute Myocardial Infarction. Cardiol. J..

[135] Matysiak R., Błaszczyk J., Obrebska A., Mejer A., Koziróg M., Kowalski J. (2014). Oxidative-reductive balance in patients after acute coronary syndrome that undergo cardiac rehabilitation. Pol. Merkur. Lek..

[136] Gheddouchi S., Mokhtari-Soulimane N., Merzouk H., Bekhti F., Soulimane F., Guermouche B., Meziane Tani A., Narce M. (2015). Low SOD Activity Is Associated with Overproduction of Peroxynitrite and Nitric Oxide in Patients with Acute Coronary Syndrome. Nitric Oxide.

[137] Noichri Y., Chalghoum A., Chkioua L., Baudin B., Ernez S., Ferchichi S., Miled A. (2013). Low Erythrocyte Catalase Enzyme Activity Is Correlated with High Serum Total Homocysteine Levels in Tunisian Patients with Acute Myocardial Infarction. Diagn. Pathol..

[138] Dragan P.D., Ivan S.B., Goran D.Z., Maja N.D., Nevena L.D., Marijana A.M., Jelena V.M., Nenad Z.J., Vladimir Z.I., Turnic T.N. (2023). The Role of Systemic Oxidative Status in Coronary Arterial and Peripheral Venous Blood of Patients with Unstable Angina Pectoris. Life.

[139] Berns S.A., Schmidt E.A., Nagirnyak O.A., Osokina A.V., Klimenkova A.V., Barbarash O.L. (2019). The role of the superoxide dismutase for predicting long-term adverse events in patients with acute coronary syndrome. Klin. Lab. Diagn..

[140] Charfeddine S., Abid L., Ali Z.B., Yousfi C., Gtif I., Hammami R., Kammoun S. (2020). Oxidative Stress and Left Ventricular Dysfunction Following Acute Coronary Syndrome. Arch. Cardiovasc. Dis. Suppl..

[141] Holley A., Pitman J., Miller J., Harding S., Larsen P. (2017). Glutathione Peroxidase Activity and Expression Levels Are Significantly Increased in Acute Coronary Syndromes. J. Investig. Med..

[142] Mohammadi A., Balizadeh Karami A.R., Dehghan Mashtani V., Sahraei T., Bandani Tarashoki Z., Khattavian E., Mobarak S., Moradi Kazerouni H., Radmanesh E. (2021). Evaluation of Oxidative Stress, Apoptosis, and Expression of MicroRNA-208a and MicroRNA-1 in Cardiovascular Patients. Rep. Biochem. Mol. Biol..

[143] Matin E., Ghaffari S., Garjani A., Roshanravan N., Matin S., Alamdari N.M., Safaie N. (2020). Oxidative Stress and Its Association with ST Resolution and Clinical Outcome Measures in Patients with ST-Segment Elevation Myocardial Infarction (STEMI) Undergoing Primary Percutaneous Coronary Intervention. BMC Res. Notes.

[144] Holley A., Miller J., Harding S., Larsen P. (2014). P776Prognostic Significance of Antioxidant Enzymes in Acute Coronary Syndromes. Cardiovasc. Res..

[145] Holley A.S., Harding S.A., Sasse A., Miller J.H., Larsen P.D. (2016). Reduced Glutathione Peroxidase Activity Predicts Increased Cardiovascular Risk Following an Acute Coronary Syndrome. Int. Cardiovasc. Forum J..

[146] Gianazza E., Brioschi M., Fernandez A.M., Banfi C. (2019). Lipoxidation in Cardiovascular Diseases. Redox Biol..

[147] Emert B., Hasin-Brumshtein Y., Springstead J.R., Vakili L., Berliner J.A., Lusis A.J. (2014). HDL Inhibits the Effects of Oxidized Phospholipids on Endothelial Cell Gene Expression via Multiple Mechanisms. J. Lipid Res..

[148] Panda P., Verma H.K., Lakkakula S., Merchant N., Kadir F., Rahman S., Jeffree M.S., Lakkakula B.V.K.S., Rao P.V. (2022). Biomarkers of Oxidative Stress Tethered to Cardiovascular Diseases. Oxid. Med. Cell Longev..

[149] Zuin M., Capatti E., Borghi C., Zuliani G. (2022). Serum Malondialdehyde Levels in Hypertensive Patients: A Non-Invasive Marker of Oxidative Stress. A Systematic Review and Meta-Analysis. High. Blood Press. Cardiovasc. Prev..

[150] Adnan M.T., Amin M.N., Uddin M.G., Hussain M.S., Sarwar M.S., Hossain M.K., Uddin S.M.N., Islam M.S. (2019). Increased Concentration of Serum MDA, Decreased Antioxidants and Altered Trace Elements and Macro-Minerals Are Linked to Obesity among Bangladeshi Population. Diabetes Metab. Syndr..

[151] Yang R.-L., Shi Y.-H., Hao G., Li W., Le G.-W. (2008). Increasing Oxidative Stress with Progressive Hyperlipidemia in Human: Relation between Malondialdehyde and Atherogenic Index. J. Clin. Biochem. Nutr..

[152] Shah A.A., Khand F., Khand T.U. (2015). Effect of Smoking on Serum Xanthine Oxidase, Malondialdehyde, Ascorbic Acid and α-Tocopherol Levels in Healthy Male Subjects. Pak. J. Med. Sci..

[153] Walter M.F., Jacob R.F., Jeffers B., Ghadanfar M.M., Preston G.M., Buch J., Mason R.P. (2004). Serum Levels of Thiobarbituric Acid Reactive Substances Predict Cardiovascular Events in Patients with Stable Coronary Artery Disease: A Longitudinal Analysis of the PREVENT Study. J. Am. Coll. Cardiol..

[154] Jaganjac M., Milkovic L., Gegotek A., Cindric M., Zarkovic K., Skrzydlewska E., Zarkovic N. (2020). The Relevance of Pathophysiological Alterations in Redox Signaling of 4-Hydroxynonenal for Pharmacological Therapies of Major Stress-Associated Diseases. Free Radic. Biol. Med..

[155] Nègre-Salvayre A., Garoby-Salom S., Swiader A., Rouahi M., Pucelle M., Salvayre R. (2017). Proatherogenic Effects of 4-Hydroxynonenal. Free Radic. Biol. Med..

[156] Bauer J., Ripperger A., Frantz S., Ergün S., Schwedhelm E., Benndorf R.A. (2014). Pathophysiology of Isoprostanes in the Cardiovascular System: Implications of Isoprostane-Mediated Thromboxane A2 Receptor Activation. Br. J. Pharmacol..

[157] Davies S.S., Roberts L.J. (2011). F2-Isoprostanes as an Indicator and Risk Factor for Coronary Heart Disease. Free Radic. Biol. Med..

[158] Heravi A.S., Zhao D., Michos E.D., Doria De Vasconcellos H., Ambale-Venkatesh B., Lloyd-Jones D., Schreiner P.J., Reis J.P., Shikany J.M., Lewis C.E. (2023). Oxidative Stress and Cardiovascular Risk Factors: The Coronary Artery Risk Development in Young Adults (CARDIA) Study. Antioxidants.

[159] Fort-Gallifa I., García-Heredia A., Hernández-Aguilera A., Simó J.M., Sepúlveda J., Martín-Paredero V., Camps J., Joven J. (2016). Biochemical Indices of Oxidative Stress and Inflammation in the Evaluation of Peripheral Artery Disease. Free Radic. Biol. Med..

[160] Kuchta A., Strzelecki A., Ćwiklińska A., Totoń M., Gruchała M., Zdrojewski Z., Kortas-Stempak B., Gliwińska A., Dąbkowski K., Jankowski M. (2015). PON-1 Activity and Plasma 8-Isoprostane Concentration in Patients with Angiographically Proven Coronary Artery Disease. Oxid. Med. Cell. Longev..

[161] Wang B., Pan J., Wang L., Zhu H., Yu R., Zou Y. (2006). Associations of Plasma 8-Isoprostane Levels with the Presence and Extent of Coronary Stenosis in Patients with Coronary Artery Disease. Atherosclerosis.

[162] Basarici I., Altekin R.E., Demir I., Yilmaz H. (2008). Urinary 8-Isoprostane Levels Can Indicate the Presence, Severity and Extent of Angiographic Coronary Artery Disease. Acta Cardiol..

[163] Schwedhelm E., Bartling A., Lenzen H., Tsikas D., Maas R., Brümmer J., Gutzki F.-M., Berger J., Frölich J.C., Böger R.H. (2004). Urinary 8-Iso-Prostaglandin F2alpha as a Risk Marker in Patients with Coronary Heart Disease: A Matched Case-Control Study. Circulation.

[164] Gołąb A., Plicner D., Rzucidło-Hymczak A., Tomkiewicz-Pająk L., Gawęda B., Kapelak B., Undas A. (2022). 8-Isoprostanes and Asymmetric Dimethylarginine as Predictors of Mortality in Patients Following Coronary Bypass Surgery: A Long-Term Follow-Up Study. J. Clin. Med..

[165] Roest M., Voorbij H.A.M., Van der Schouw Y.T., Peeters P.H.M., Teerlink T., Scheffer P.G. (2008). High Levels of Urinary F2-Isoprostanes Predict Cardiovascular Mortality in Postmenopausal Women. J. Clin. Lipidol..

[166] Gao S., Zhao D., Qi Y., Wang W., Wang M., Sun J., Liu J., Li Y., Liu J. (2018). Circulating Oxidized Low-Density Lipoprotein Levels Independently Predict 10-Year Progression of Subclinical Carotid Atherosclerosis: A Community-Based Cohort Study. J. Atheroscler. Thromb..

[167] Lopes-Virella M.F., Hunt K.J., Baker N.L., Moritz T., Virella G. (2012). The Levels of MDA—LDL in Circulating Immune Complexes Predict Myocardial Infarction in the VADT Study. Atherosclerosis.

[168] Tsimikas S., Willeit P., Willeit J., Santer P., Mayr M., Xu Q., Mayr A., Witztum J.L., Kiechl S. (2012). Oxidation-Specific Biomarkers, Prospective 15-Year Cardiovascular and Stroke Outcomes, and Net Reclassification of Cardiovascular Events. J. Am. Coll. Cardiol..

[169] Nishi K., Itabe H., Uno M., Kitazato K.T., Horiguchi H., Shinno K., Nagahiro S. (2002). Oxidized LDL in Carotid Plaques and Plasma Associates with Plaque Instability. Arterioscler. Thromb. Vasc. Biol..

[170] Prasad A., Clopton P., Ayers C., Khera A., de Lemos J.A., Witztum J.L., Tsimikas S. (2017). Relationship of Autoantibodies to MDA-LDL and ApoB-Immune Complexes to Sex, Ethnicity, Subclinical Atherosclerosis, and Cardiovascular Events. Arterioscler. Thromb. Vasc. Biol..

[171] Xu L., Yan X., Tang Z., Feng B. (2022). Association between Circulating Oxidized OxLDL/LDL-C Ratio and the Severity of Coronary Atherosclerosis, along with Other Emerging Biomarkers of Cardiovascular Disease in Patients with Type 2 Diabetes. Diabetes Res. Clin. Pract..

[172] Zhang Q., Ai Y., Dong H., Wang J., Xu L. (2019). Circulating Oxidized Low-Density Lipoprotein Is a Strong Risk Factor for the Early Stage of Coronary Heart Disease. IUBMB Life.

[173] Moohebati M., Kabirirad V., Ghayour-Mobarhan M., Esmaily H., Tavallaie S., Akhavan Rezayat A., Pourghadamyari H., Sahebkar A. (2014). Investigation of Serum Oxidized Low-Density Lipoprotein IgG Levels in Patients with Angiographically Defined Coronary Artery Disease. Int. J. Vasc. Med..

[174] Sevinc Ok E., Kircelli F., Asci G., Altunel E., Ertilav M., Sipahi S., Bozkurt D., Duman S., Ozkahya M., Toz H. (2012). Neither Oxidized nor Anti-Oxidized Low-Density Lipoprotein Level Is Associated with Atherosclerosis or Mortality in Hemodialysis Patients. Hemodial. Int..

[175] van den Berg V.J., Haskard D.O., Fedorowski A., Hartley A., Kardys I., Caga-Anan M., Akkerhuis K.M., Oemrawsingh R.M., van Geuns R.J., de Jaegere P. (2018). IgM Anti-Malondialdehyde Low Density Lipoprotein Antibody Levels Indicate Coronary Heart Disease and Necrotic Core Characteristics in the Nordic Diltiazem (NORDIL) Study and the Integrated Imaging and Biomarker Study 3 (IBIS-3). eBioMedicine.

[176] Khamis R.Y., Hughes A.D., Caga-Anan M., Chang C.L., Boyle J.J., Kojima C., Welsh P., Sattar N., Johns M., Sever P. (2016). High Serum Immunoglobulin G and M Levels Predict Freedom From Adverse Cardiovascular Events in Hypertension: A Nested Case-Control Substudy of the Anglo-Scandinavian Cardiac Outcomes Trial. eBioMedicine.

[177] Ehara S., Ueda M., Naruko T., Haze K., Itoh A., Otsuka M., Komatsu R., Matsuo T., Itabe H., Takano T. (2001). Elevated Levels of Oxidized Low Density Lipoprotein Show a Positive Relationship with the Severity of Acute Coronary Syndromes. Circulation.

[178] Medeiros A.M.B., von Mühlen C.A., Gidlund M.A., Bodanese R., Gottlieb M.G.V., Bodanese L.C. (2010). Antibodies against oxLDL and Acute Coronary Syndrome. Arq. Bras. Cardiol..

[179] Gruzdeva O., Uchasova E., Dyleva Y., Belik E., Karetnikova V., Shilov A., Barbarash O. (2014). Multivessel Coronary Artery Disease, Free Fatty Acids, Oxidized LDL and Its Antibody in Myocardial Infarction. Lipids Health Dis..

[180] Zhang Y., Tang Y., Chen Y., Huang X., Zhang M., Chen J., Sun Y., Li Y. (2014). Oxidized Low-Density Lipoprotein and C-Reactive Protein Have Combined Utility for Better Predicting Prognosis after Acute Coronary Syndrome. Cell. Biochem. Biophys..

[181] Shahzad S., Hasan A., Faizy A.F., Mateen S., Fatima N., Moin S. (2018). Elevated DNA Damage, Oxidative Stress, and Impaired Response Defense System Inflicted in Patients With Myocardial Infarction. Clin. Appl. Thromb. Hemost..

[182] Kosek V., Hajšl M., Bechyňská K., Kučerka O., Suttnar J., Hlaváčková A., Hajšlová J., Malý M. (2022). Long-Term Effects on the Lipidome of Acute Coronary Syndrome Patients. Metabolites.

[183] Barrios M.J., Muñoz G., Torres R., Bueno Y. (2012). 4-Hydroxynonenal as Oxidative Damage Index in Acute Myocardial Infarction. Free Radic. Biol. Med..

[184] Leonarduzzi G., Gargiulo S., Rossin D., Testa G., Gamba P., Staurenghi E., Giannelli S., Sottero B., Biasi F., Poli G. (2018). Oxidized Lipids in Atherosclerotic Plaque Instability. Free Radic. Biol. Med..

[185] Nishibe A., Kijima Y., Fukunaga M., Nishiwaki N., Sakai T., Nakagawa Y., Hata T. (2008). Increased Isoprostane Content in Coronary Plaques Obtained from Vulnerable Patients. Prostaglandins Leukot. Essent. Fat. Acids.

[186] Vichova T., Waldauf P., Karpisek M., Jarkovsky J., Motovska Z. (2021). Oxidative Stress Markers, Thioredoxin 1 and 8-Isoprostane, in Relation to Ischemic Time in Patients with ST-segment Elevation Myocardial Infarction Treated by Primary Percutaneous Coronary Intervention. Pol. Arch. Intern. Med..

[187] Undas A., Szułdrzynski K., Stepien E., Zalewski J., Godlewski J., Tracz W., Pasowicz M., Zmudka K. (2008). Reduced Clot Permeability and Susceptibility to Lysis in Patients with Acute Coronary Syndrome: Effects of Inflammation and Oxidative Stress. Atherosclerosis.

[188] Szułdrzyński K., Zalewski J., Machnik A., Zmudka K. (2010). Elevated Levels of 8-Iso-Prostaglandin F2alpha in Acute Coronary Syndromes Are Associated with Systemic and Local Platelet Activation. Pol. Arch. Med. Wewn..

[189] Reilly M.P., Delanty N., Roy L., Rokach J., Callaghan P.O., Crean P., Lawson J.A., FitzGerald G.A. (1997). Increased Formation of the Isoprostanes IPF2alpha-I and 8-Epi-Prostaglandin F2alpha in Acute Coronary Angioplasty: Evidence for Oxidant Stress during Coronary Reperfusion in Humans. Circulation.

[190] LeLeiko R.M., Vaccari C.S., Sola S., Merchant N., Nagamia S.H., Thoenes M., Khan B.V. (2009). Usefulness of Elevations in Serum Choline and Free F_2_-Isoprostane to Predict 30-Day Cardiovascular Outcomes in Patients with Acute Coronary Syndrome. Am. J. Cardiol..

[191] Rashdan N.A., Shrestha B., Pattillo C.B. (2020). S-Glutathionylation, Friend or Foe in Cardiovascular Health and Disease. Redox Biol..

[192] Wang Y., Qiao M., Mieyal J.J., Asmis L.M., Asmis R. (2006). Molecular Mechanism of Glutathione-Mediated Protection from Oxidized Low-Density Lipoprotein-Induced Cell Injury in Human Macrophages: Role of Glutathione Reductase and Glutaredoxin. Free Radic. Biol. Med..

[193] Nonaka K., Kume N., Urata Y., Seto S., Kohno T., Honda S., Ikeda S., Muroya T., Ikeda Y., Ihara Y. (2007). Serum Levels of S-Glutathionylated Proteins as a Risk-Marker for Arteriosclerosis Obliterans. Circ. J..

[194] Valerio V., Myasoedova V.A., Moschetta D., Porro B., Perrucci G.L., Cavalca V., Cavallotti L., Songia P., Poggio P. (2019). Impact of Oxidative Stress and Protein S-Glutathionylation in Aortic Valve Sclerosis Patients with Overt Atherosclerosis. J. Clin. Med..

[195] Sánchez G., Pedrozo Z., Domenech R.J., Hidalgo C., Donoso P. (2005). Tachycardia Increases NADPH Oxidase Activity and RyR2 S-Glutathionylation in Ventricular Muscle. J. Mol. Cell. Cardiol..

[196] Lancel S., Zhang J., Evangelista A., Trucillo M.P., Tong X., Siwik D.A., Cohen R.A., Colucci W.S. (2009). Nitroxyl Activates SERCA in Cardiac Myocytes via Glutathiolation of Cysteine 674. Circ. Res..

[197] Bartesaghi S., Radi R. (2018). Fundamentals on the Biochemistry of Peroxynitrite and Protein Tyrosine Nitration. Redox Biol..

[198] He C., Choi H.C., Xie Z. (2010). Enhanced Tyrosine Nitration of Prostacyclin Synthase Is Associated with Increased Inflammation in Atherosclerotic Carotid Arteries from Type 2 Diabetic Patients. Am. J. Pathol..

[199] Medeiros R., Sousa B., Rossi S., Afonso C., Bonino L., Pitt A., López E., Spickett C., Borthagaray G. (2021). Identification and Relative Quantification of 3-Nitrotyrosine Residues in Fibrinogen Nitrated in Vitro and Fibrinogen from Ischemic Stroke Patient Plasma Using LC-MS/MS. Free Radic. Biol. Med..

[200] Thomson L., Tenopoulou M., Lightfoot R., Tsika E., Parastatidis I., Martinez M., Greco T.M., Doulias P.-T., Wu Y., Tang W.H.W. (2012). Immunoglobulins against Tyrosine-Nitrated Epitopes in Coronary Artery Disease. Circulation.

[201] Pourfarzam M., Movahedian A., Sarrafzadegan N., Basati G., Samsamshariat S.Z. (2013). Association between Plasma Myeloperoxidase and Free 3-Nitrotyrosine Levels in Patients with Coronary Artery Disease. Int. J. Clin. Med..

[202] Jørgensen S.M., Lorentzen L.G., Hammer A., Hoefler G., Malle E., Chuang C.Y., Davies M.J. (2023). The Inflammatory Oxidant Peroxynitrous Acid Modulates the Structure and Function of the Recombinant Human V3 Isoform of the Extracellular Matrix Proteoglycan Versican. Redox Biol..

[203] Colombo G., Clerici M., Garavaglia M.E., Giustarini D., Rossi R., Milzani A., Dalle-Donne I. (2016). A Step-by-Step Protocol for Assaying Protein Carbonylation in Biological Samples. J. Chromatogr. B Anal. Technol. Biomed. Life Sci..

[204] Yuan Q., Zhu X., Sayre L.M. (2007). Chemical Nature of Stochastic Generation of Protein-Based Carbonyls: Metal-Catalyzed Oxidation versus Modification by Products of Lipid Oxidation. Chem. Res. Toxicol..

[205] Pirinccioglu A.G., Gökalp D., Pirinccioglu M., Kizil G., Kizil M. (2010). Malondialdehyde (MDA) and Protein Carbonyl (PCO) Levels as Biomarkers of Oxidative Stress in Subjects with Familial Hypercholesterolemia. Clin. Biochem..

[206] Sigala F., Kotsinas A., Savari P., Filis K., Markantonis S., Iliodromitis E.K., Gorgoulis V.G., Andreadou I. (2010). Oxidized LDL in Human Carotid Plaques Is Related to Symptomatic Carotid Disease and Lesion Instability. J. Vasc. Surg..

[207] Kamruzzaman M., Choudhury T.Z., Rahman T., Islam L.N. (2019). A Cross-Sectional Study on Assessment of Oxidative Stress in Coronary Heart Disease Patients in Bangladesh. World J. Cardiovasc. Dis..

[208] Tejaswi G., Suchitra M.M., Rajasekhar D., Kiranmayi V.S., Rao P.S. (2017). Myeloperoxidase, Protein Carbonyls and Oxidative Stress in Coronary Artery Disease. J. Indian Coll. Cardiol..

[209] Cournot M., Burillo E., Saulnier P., Planesse C., Gand E., Rehman M., Ragot S., Rondeau P., Catan A., Gonthier M. (2018). Circulating Concentrations of Redox Biomarkers Do Not Improve the Prediction of Adverse Cardiovascular Events in Patients With Type 2 Diabetes Mellitus. J. Am. Heart Assoc..

[210] Amrita J., Mahajan M., Bhanwer A.J.S., Mohan G. (2016). Oxidative Stress: An Effective Prognostic Tool for an Early Detection of Cardiovascular Disease in Menopausal Women. Biochem. Res. Int..

[211] Shevtsova A., Gordiienko I., Tkachenko V., Ushakova G. (2021). Ischemia-Modified Albumin: Origins and Clinical Implications. Dis. Markers.

[212] Uslu A.U., Kucuk A., Balta S., Ozturk C., Arslan S., Tekin L., Kucuksen S., Toker A., Kayrak M. (2019). The Relation between Ischemia Modified Albumin Levels and Carotid Intima Media Thickness in Patients with Rheumatoid Arthritis. Int. J. Rheum. Dis..

[213] Xiao J., Lu Y., Yang X. (2020). Ultrasound Detection of Epicardial Adipose Tissue Combined With Ischemic Modified Albumin in the Diagnosis of Coronary Heart Disease. Heart Surg. Forum.

[214] Zhong Y., Wang N., Xu H., Hou X., Xu P., Zhou Z. (2012). Ischemia-Modified Albumin in Stable Coronary Atherosclerotic Heart Disease: Clinical Diagnosis and Risk Stratification. Coron. Artery Dis..

[215] Su X., Zhang K., Guo F., Yuan B., Wang C., Xiao L., Wang J., Huang H. (2013). Ischemia-Modified Albumin, a Predictive Marker of Major Adverse Cardiovascular Events in Continuous Ambulatory Peritoneal Dialysis Patients. Clin. Biochem..

[216] Eaton P., Wright N., Hearse D.J., Shattock M.J. (2002). Glyceraldehyde Phosphate Dehydrogenase Oxidation during Cardiac Ischemia and Reperfusion. J. Mol. Cell. Cardiol..

[217] Chen F.C., Ogut O. (2006). Decline of Contractility during Ischemia-Reperfusion Injury: Actin Glutathionylation and Its Effect on Allosteric Interaction with Tropomyosin. Am. J. Physiol. Cell. Physiol..

[218] Wang K., Hirschenson J., Moore A., Mailloux R.J. (2022). Conditions Conducive to the Glutathionylation of Complex I Subunit NDUFS1 Augment ROS Production Following the Oxidation of Ubiquinone Linked Substrates, Glycerol-3-Phosphate and Proline. Antioxidants.

[219] Dominguez-Rodriguez A., Abreu-Gonzalez P., Consuegra-Sanchez L., Avanzas P., Sanchez-Grande A., Conesa-Zamora P. (2016). Thrombus Aspirated from Patients with ST-Elevation Myocardial Infarction: Association between 3-Nitrotyrosine and Inflammatory Markers—Insights from ARTERIA Study. Int. J. Med. Sci..

[220] Goulart A., Santos I.S., Sitnik D., Staniak H.L., Fedeli L.M., Pastore C.A., Samesima N., Bittencourt M.S., Pereira A.C., Lotufo P.A. (2013). Design and Baseline Characteristics of a Coronary Heart Disease Prospective Cohort: Two-Year Experience from the Strategy of Registry of Acute Coronary Syndrome Study (ERICO Study). Clinicsc.

[221] Quidim A.V.L., Bruno T., Leocádio P.C.L., Santos I.S., Alvarez-Leite J.I., Dos Reis Menta P.L., Lotufo P.A., Benseñor I.M., Goulart A.C. (2019). The Prognostic Value of Nitrotyrosine Levels in Coronary Heart Disease: Long-Term Evaluation in the Acute Coronary Syndrome Registry Strategy (ERICO Study). Clin. Biochem..

[222] Binti N.N., Ferdausi N., Anik M.E.K., Islam L.N. (2022). Association of Albumin, Fibrinogen, and Modified Proteins with Acute Coronary Syndrome. PLoS ONE.

[223] Gholikhani-Darbroud R., Khaki-Khatibi F. (2018). Increased Circulatory Levels of Ischemia Modified Albumin, Protein Carbonyl, Malondialdehyde and Total Antioxidant Capacity as Prognostic Biomarkers for Non-ST-Segment Elevation Myocardial Infarction: A ROC Curve Analysis. Crescent J. Med. Biol. Sci..

[224] Paton L.N., Mocatta T.J., Richards A.M., Winterbourn C.C. (2010). Increased Thrombin-Induced Polymerization of Fibrinogen Associated with High Protein Carbonyl Levels in Plasma from Patients Post Myocardial Infarction. Free Radic. Biol. Med..

[225] Mishra B., Pandey S., Niraula S.R., Rai B.K., Karki P., Baral N., Lamsal M. (2018). Utility of Ischemia Modified Albumin as an Early Marker for Diagnosis of Acute Coronary Syndrome. J. Nepal. Health Res. Counc..

[226] Jawade P., Khillare K.M., Mangudkar S., Palange A., Dhadwad J., Deshmukh M. (2023). A Comparative Study of Ischemia-Modified Albumin: A Promising Biomarker for Early Detection of Acute Coronary Syndrome (ACS). Cureus.

[227] Gurumurthy P., Borra S.K., Yeruva R.K.R., Victor D., Babu S., Cherian K.M. (2014). Estimation of Ischemia Modified Albumin (IMA) Levels in Patients with Acute Coronary Syndrome. Indian J. Clin. Biochem..

[228] Mehta M.D., Marwah S.A., Ghosh S., Shah H.N., Trivedi A.P., Haridas N. (2015). A Synergistic Role of Ischemia Modified Albumin and High-Sensitivity Troponin T in the Early Diagnosis of Acute Coronary Syndrome. J. Fam. Med. Prim. Care.

[229] Turan T., Akyüz A.R., Sahin S., Kul S., Yilmaz A.S., Kara F., Mentese S.O., Aykan A.Ç., Demir S., Celik S. (2017). Association between the Plasma Levels of IMA and Coronary Atherosclerotic Plaque Burden and Ischemic Burden in Early Phase of Non-ST-Segment-Elevation Acute Coronary Syndromes. Eur. Rev. Med. Pharmacol. Sci..

[230] Dominguez-Rodriguez A., Kaski J.C., Abreu-Gonzalez P., Samimi-Fard S. (2009). Role of Ischemia Modified Albumin to ST-Segment Resolution after Mechanical Reperfusion in Patients with ST-Segment Elevation Myocardial Infarction. Atherosclerosis.

[231] Van Belle E., Dallongeville J., Vicaut E., Degrandsart A., Baulac C., Montalescot G., OPERA Investigators (2010). Ischemia-Modified Albumin Levels Predict Long-Term Outcome in Patients with Acute Myocardial Infarction. The French Nationwide OPERA Study. Am. Heart J..

[232] Bhagavan N.V., Lai E.M., Rios P.A., Yang J., Ortega-Lopez A.M., Shinoda H., Honda S.A.A., Rios C.N., Sugiyama C.E., Ha C.-E. (2003). Evaluation of Human Serum Albumin Cobalt Binding Assay for the Assessment of Myocardial Ischemia and Myocardial Infarction. Clin. Chem..

[233] Shin H., Kim J.-G., Jang B.-H., Lim T.-H., Kim W., Cho Y., Choi K.-S., Na M.-K., Ahn C., Lee J. (2022). Diagnostic Accuracy of Ischemia-Modified Albumin for Acute Coronary Syndrome: A Systematic Review and Meta-Analysis. Medicina.

[234] Larsen E.L., Weimann A., Poulsen H.E. (2019). Interventions Targeted at Oxidatively Generated Modifications of Nucleic Acids Focused on Urine and Plasma Markers. Free Radic. Biol. Med..

[235] Martinet W., Knaapen M.W.M., De Meyer G.R.Y., Herman A.G., Kockx M.M. (2002). Elevated Levels of Oxidative DNA Damage and DNA Repair Enzymes in Human Atherosclerotic Plaques. Circulation.

[236] Zhao Y., Liang W., Tian S., Shen L., Yang H. (2020). Impact of Increased Serum 8-Hydroxy-2′-Deoxyguanosine Levels on Extent of Coronary Artery Lesions in Elderly Patients with Type 2 Diabetes. J. Int. Med. Res..

[237] Shah A., Gray K., Figg N., Finigan A., Starks L., Bennett M. (2018). Defective Base Excision Repair of Oxidative DNA Damage in Vascular Smooth Muscle Cells Promotes Atherosclerosis. Circulation.

[238] Wang X.-B., Cui N.-H., Liu X., Liu X. (2020). Mitochondrial 8-Hydroxy-2’-Deoxyguanosine and Coronary Artery Disease in Patients with Type 2 Diabetes Mellitus. Cardiovasc. Diabetol..

[239] Broedbaek K., Køster-Rasmussen R., Siersma V., Persson F., Poulsen H.E., de Fine Olivarius N. (2017). Urinary Albumin and 8-Oxo-7,8-Dihydroguanosine as Markers of Mortality and Cardiovascular Disease during 19 Years after Diagnosis of Type 2 Diabetes—A Comparative Study of Two Markers to Identify High Risk Patients. Redox Biol..

[240] Xuan Y., Gào X., Holleczek B., Brenner H., Schöttker B. (2018). Prediction of Myocardial Infarction, Stroke and Cardiovascular Mortality with Urinary Biomarkers of Oxidative Stress: Results from a Large Cohort Study. Int. J. Cardiol..

[241] Di Minno A., Turnu L., Porro B., Squellerio I., Cavalca V., Tremoli E., Di Minno M.N.D. (2016). 8-Hydroxy-2-Deoxyguanosine Levels and Cardiovascular Disease: A Systematic Review and Meta-Analysis of the Literature. Antioxid. Redox Signal..

[242] Arca M., Conti B., Montali A., Pignatelli P., Campagna F., Barillà F., Tanzilli G., Verna R., Vestri A., Gaudio C. (2008). C242T Polymorphism of NADPH Oxidase P22phox and Recurrence of Cardiovascular Events in Coronary Artery Disease. Arterioscler. Thromb. Vasc. Biol..

[243] Vukajlovic J.T., Simic I., Milosevic-Djordjevic O. (2021). DNA and Chromosomal Damage in Peripheral Blood Lymphocytes in Patients with Acute Coronary Syndrome Undergoing a Coronary Angiography. Anatol. J. Cardiol..

[244] Bliksøen M., Baysa A., Eide L., Bjørås M., Suganthan R., Vaage J., Stensløkken K.O., Valen G. (2015). Mitochondrial DNA Damage and Repair during Ischemia-Reperfusion Injury of the Heart. J. Mol. Cell. Cardiol..

[245] Himmetoglu S., Dincer Y., Bozcali E., Ali Vural V., Akcay T. (2009). Oxidative DNA Damage and Antioxidant Defense after Reperfusion in Acute Myocardial Infarction. J. Investig. Med..

[246] Gohbara M., Iwahashi N., Nakahashi H., Kataoka S., Takahashi H., Kirigaya J., Minamimoto Y., Akiyama E., Okada K., Matsuzawa Y. (2021). Clinical Impact of Admission Urinary 8-Hydroxydeoxyguanosine Level for Predicting Cardiovascular Mortality in Patients with Acute Coronary Syndrome. Heart Vessel..

[247] Tomandlova M., Parenica J., Lokaj P., Ondrus T., Kala P., Miklikova M., Helanova K., Helan M., Malaska J., Benesova K. (2021). Prognostic Value of Oxidative Stress in Patients with Acute Myocardial Infarction Complicated by Cardiogenic Shock: A Prospective Cohort Study. Free Radic. Biol. Med..

[248] Jin Y., Qiu C., Zheng Q., Liu L., Liu Z., Wang Y. (2015). Efficacy of Different Doses of Atorvastatin Treatment on Serum Levels of 8-Hydroxy-Guanin (8-OHdG) and Cardiac Function in Patients with Ischemic Cardiomyopathy. Pak. J. Med. Sci..

[249] Aladağ N., Asoğlu R., Ozdemir M., Asoğlu E., Derin A.R., Demir C., Demir H. (2021). Oxidants and Antioxidants in Myocardial Infarction (MI): Investigation of Ischemia Modified Albumin, Malondialdehyde, Superoxide Dismutase and Catalase in Individuals Diagnosed with ST Elevated Myocardial Infarction (STEMI) and Non-STEMI (NSTEMI). J. Med. Biochem..

[250] Inoue T., Uchida T., Kamishirado H., Takayanagi K., Hayashi T., Morooka S. (2001). Clinical Significance of Antibody against Oxidized Low Density Lipoprotein in Patients with Atherosclerotic Coronary Artery Disease. J. Am. Coll. Cardiol..

[251] Yaghoubi A., Ghojazadeh M., Abolhasani S., Alikhah H., Khaki-Khatibi F. (2015). Correlation of Serum Levels of Vitronectin, Malondialdehyde and Hs- CRP With Disease Severity in Coronary Artery Disease. J. Cardiovasc. Thorac. Res..

[252] Shoeb M., Ansari N.H., Srivastava S.K., Ramana K.V. (2014). 4-Hydroxynonenal in the Pathogenesis and Progression of Human Diseases. Curr. Med. Chem..

[253] Gross M., Steffes M., Jacobs D.R., Yu X., Lewis L., Lewis C.E., Loria C.M. (2005). Plasma F2-Isoprostanes and Coronary Artery Calcification: The CARDIA Study. Clin. Chem..

[254] Mueller T., Dieplinger B., Gegenhuber A., Haidinger D., Schmid N., Roth N., Ebner F., Landl M., Poelz W., Haltmayer M. (2004). Serum Total 8-Iso-Prostaglandin F2alpha: A New and Independent Predictor of Peripheral Arterial Disease. J. Vasc. Surg..

[255] Taylor E.R., Hurrell F., Shannon R.J., Lin T.-K., Hirst J., Murphy M.P. (2003). Reversible Glutathionylation of Complex I Increases Mitochondrial Superoxide Formation. J. Biol. Chem..

[256] Mu H., Wang X., Lin P., Yao Q., Chen C. (2008). Nitrotyrosine Promotes Human Aortic Smooth Muscle Cell Migration through Oxidative Stress and ERK1/2 Activation. Biochim. Biophys. Acta.

[257] Caimi G., Canino B., Incalcaterra E., Ferrera E., Montana M., Lo Presti R. (2013). Behaviour of Protein Carbonyl Groups in Juvenile Myocardial Infarction. Clin. Hemorheol. Microcirc..

[258] Strauss M.H., Hall A.S., Narkiewicz K. (2021). The Combination of Beta-Blockers and ACE Inhibitors Across the Spectrum of Cardiovascular Diseases. Cardiovasc. Drugs Ther..

[259] Wu H.-P., Yang F.-C., Lin H.-D., Cai C.-Z., Chuang M.-J., Chiang K.F., Lin M.-J. (2024). Association between Statin Therapy and Long-Term Clinical Outcomes in Patients with Stable Coronary Disease Undergoing Percutaneous Coronary Intervention. Sci. Rep..

[260] Vrints C., Andreotti F., Koskinas K.C., Rossello X., Adamo M., Ainslie J., Banning A.P., Budaj A., Buechel R.R., Chiariello G.A. (2024). 2024 ESC Guidelines for the Management of Chronic Coronary Syndromes. Eur. Heart J..

[261] Zinellu A., Mangoni A.A. (2021). A Systematic Review and Meta-Analysis of the Effect of Statins on Glutathione Peroxidase, Superoxide Dismutase, and Catalase. Antioxidants.

[262] Zhang X., Ding M., Zhu P., Huang H., Zhuang Q., Shen J., Cai Y., Zhao M., He Q. (2019). New Insights into the Nrf-2/HO-1 Signaling Axis and Its Application in Pediatric Respiratory Diseases. Oxid. Med. Cell. Longev..

[263] Tong H., Zhang X., Meng X., Lu L., Mai D., Qu S. (2018). Simvastatin Inhibits Activation of NADPH Oxidase/P38 MAPK Pathway and Enhances Expression of Antioxidant Protein in Parkinson Disease Models. Front. Mol. Neurosci..

[264] Moon G.J., Kim S.J., Cho Y.H., Ryoo S., Bang O.Y. (2014). Antioxidant Effects of Statins in Patients with Atherosclerotic Cerebrovascular Disease. J. Clin. Neurol..

[265] Jian Z., Tang L., Yi X., Liu B., Zhang Q., Zhu G., Wang G., Gao T., Li C. (2016). Aspirin Induces Nrf2-Mediated Transcriptional Activation of Haem Oxygenase-1 in Protection of Human Melanocytes from H_2_ O_2_ -Induced Oxidative Stress. J. Cell. Mol. Med..

[266] Chen C.-M., Tung Y.-T., Wei C.-H., Lee P.-Y., Chen W. (2020). Anti-Inflammatory and Reactive Oxygen Species Suppression through Aspirin Pretreatment to Treat Hyperoxia-Induced Acute Lung Injury in NF-κB–Luciferase Inducible Transgenic Mice. Antioxidants.

[267] Ayyadevara S., Bharill P., Dandapat A., Hu C., Khaidakov M., Mitra S., Shmookler Reis R.J., Mehta J.L. (2013). Aspirin Inhibits Oxidant Stress, Reduces Age-Associated Functional Declines, and Extends Lifespan of Caenorhabditis Elegans. Antioxid. Redox Signal..

[268] Kurban S., Mehmetoglu I. (2010). Effects of Acetylsalicylic Acid on Serum Paraoxonase Activity, Ox-LDL, Coenzyme Q10 and Other Oxidative Stress Markers in Healthy Volunteers. Clin. Biochem..

[269] Berg K., Langaas M., Ericsson M., Pleym H., Basu S., Nordrum I.S., Vitale N., Haaverstad R. (2013). Acetylsalicylic Acid Treatment until Surgery Reduces Oxidative Stress and Inflammation in Patients Undergoing Coronary Artery Bypass Grafting. Eur. J. Cardiothorac. Surg..

[270] Pradhan A., Tiwari A., Caminiti G., Salimei C., Muscoli S., Sethi R., Perrone M.A. (2022). Ideal P2Y12 Inhibitor in Acute Coronary Syndrome: A Review and Current Status. Int. J. Environ. Res. Public. Health.

[271] El-Mokadem B.M., El-Abhar H.S., Abdallah D.M., Awad A.S., Soubh A.A. (2021). Epac-1/Rap-1 Signaling Pathway Orchestrates the Reno-Therapeutic Effect of Ticagrelor against Renal Ischemia/Reperfusion Model. Biomed. Pharmacother..

[272] Yang H., Zhao P., Tian S. (2016). Clopidogrel Protects Endothelium by Hindering TNFα-Induced VCAM-1 Expression through CaMKKβ/AMPK/Nrf2 Pathway. J. Diabetes Res..

[273] Rudolph T.K., Fuchs A., Klinke A., Schlichting A., Friedrichs K., Hellmich M., Mollenhauer M., Schwedhelm E., Baldus S., Rudolph V. (2017). Prasugrel as Opposed to Clopidogrel Improves Endothelial Nitric Oxide Bioavailability and Reduces Platelet-Leukocyte Interaction in Patients with Unstable Angina Pectoris: A Randomized Controlled Trial. Int. J. Cardiol..

[274] Taher M.A., Nassir E.S. (2011). Beneficial Effects of Clopidogrel on Glycemic Indices and Oxidative Stress in Patients with Type 2 Diabetes. Saudi Pharm. J..

[275] Campo G., Dalla Sega F.V., Pavasini R., Aquila G., Gallo F., Fortini F., Tonet E., Cimaglia P., Del Franco A., Pestelli G. (2017). Biological Effects of Ticagrelor over Clopidogrel in Patients with Stable Coronary Artery Disease and Chronic Obstructive Pulmonary Disease. Thromb. Haemost..

[276] Kumar A., Lutsey P.L., Peter W.L.S., Schommer J.C., Hof J.R.V., Rajpurohit A., Farley J.F. (2022). Comparative Effectiveness of Ticagrelor, Prasugrel, and Clopidogrel for Secondary Prophylaxis in Acute Coronary Syndrome: A Propensity Score-Matched Cohort Study. Clin. Pharmacol. Ther..

[277] Dandona P., Ghanim H., Brooks D.P. (2007). Antioxidant Activity of Carvedilol in Cardiovascular Disease. J. Hypertens..

[278] Kowalski J., Banach M., Barylski M., Irzmanski R., Pawlicki L. (2008). Carvedilol Modifies Antioxidant Status of Patients with Stable Angina. Cell. Mol. Biol. Lett..

[279] Dandona P., Karne R., Ghanim H., Hamouda W., Aljada A., Magsino C.H. (2000). Carvedilol Inhibits Reactive Oxygen Species Generation by Leukocytes and Oxidative Damage to Amino Acids. Circulation.

[280] Wu T.-C., Chen Y.-H., Leu H.-B., Chen Y.-L., Lin F.-Y., Lin S.-J., Chen J.-W. (2007). Carvedilol, a Pharmacological Antioxidant, Inhibits Neointimal Matrix Metalloproteinase-2 and-9 in Experimental Atherosclerosis. Free Radic. Biol. Med..

[281] Oliveira P.J., Esteves T., Rolo A.P., Palmeira C.M., Moreno A.J.M. (2004). Carvedilol Inhibits the Mitochondrial Permeability Transition by an Antioxidant Mechanism. Cardiovasc. Toxicol..

[282] Karabacak M., Dogan A., Tayyar S., Bas H.A. (2015). The Effects of Carvedilol and Nebivolol on Oxidative Stress Status in Patients with Non-Ischaemic Heart Failure. Kardiol. Pol..

[283] Evangelista S., Garbin U., Pasini A.F., Stranieri C., Boccioletti V., Cominacini L. (2007). Effect of DL-Nebivolol, Its Enantiomers and Metabolites on the Intracellular Production of Superoxide and Nitric Oxide in Human Endothelial Cells. Pharmacol. Res..

[284] Fratta Pasini A., Garbin U., Nava M.C., Stranieri C., Davoli A., Sawamura T., Lo Cascio V., Cominacini L. (2005). Nebivolol Decreases Oxidative Stress in Essential Hypertensive Patients and Increases Nitric Oxide by Reducing Its Oxidative Inactivation. J. Hypertens..

[285] Serg M., Kampus P., Kals J., Zagura M., Zilmer M., Zilmer K., Kullisaar T., Eha J. (2012). Nebivolol and Metoprolol: Long-Term Effects on Inflammation and Oxidative Stress in Essential Hypertension. Scand. J. Clin. Lab. Investig..

[286] Bryniarski P., Nazimek K., Marcinkiewicz J. (2022). Immunomodulatory Activity of the Most Commonly Used Antihypertensive Drugs—Angiotensin Converting Enzyme Inhibitors and Angiotensin II Receptor Blockers. Int. J. Mol. Sci..

[287] Ushkalova V.N., Zhuravleva L.A. (2006). Kinetic Investigation of the Antioxidant Properties of Captopril. Pharm. Chem. J..

[288] Elfowiris A., Banigesh A. (2022). Evaluation of Antioxidant Therapeutic Value of ACE Inhibitor as Adjunct Therapy on Type 2 Diabetes Mellitus Patients with Cardiovascular Disease. ACS Pharmacol. Transl. Sci..

[289] Dumitrescu M., Costache G., Constantin A., Popov D. (2013). Zofenopril Functions as Antioxidant, Correcting the Renal Oxidative Damages in a Rat Model of L-Name Induced Hypertension. Ann. Rom. Soc. Cell. Biol..

[290] Napoli C., Sica V., de Nigris F., Pignalosa O., Condorelli M., Ignarro L.J., Liguori A. (2004). Sulfhydryl Angiotensin-Converting Enzyme Inhibition Induces Sustained Reduction of Systemic Oxidative Stress and Improves the Nitric Oxide Pathway in Patients with Essential Hypertension. Am. Heart J..

[291] Napoli C., Bruzzese G., Ignarro L.J., Crimi E., de Nigris F., Williams-Ignarro S., Libardi S., Sommese L., Fiorito C., Mancini F.P. (2008). Long-Term Treatment with Sulfhydryl Angiotensin-Converting Enzyme Inhibition Reduces Carotid Intima-Media Thickening and Improves the Nitric Oxide/Oxidative Stress Pathways in Newly Diagnosed Patients with Mild to Moderate Primary Hypertension. Am. Heart J..

[292] Pasini A.F., Garbin U., Nava M.C., Stranieri C., Pellegrini M., Boccioletti V., Luchetta M.L., Fabrizzi P., Lo Cascio V., Cominacini L. (2007). Effect of Sulfhydryl and Non-Sulfhydryl Angiotensin-Converting Enzyme Inhibitors on Endothelial Function in Essential Hypertensive Patients. Am. J. Hypertens..

[293] Ivanov M., Mihailović-Stanojević N., Grujić Milanović J., Jovović Đ., Marković-Lipkovski J., Ćirović S., Miloradović Z. (2014). Losartan Improved Antioxidant Defense, Renal Function and Structure of Postischemic Hypertensive Kidney. PLoS ONE.

[294] Kamper M., Tsimpoukidi O., Chatzigeorgiou A., Lymberi M., Kamper E.F. (2010). The Antioxidant Effect of Angiotensin II Receptor Blocker, Losartan, in Streptozotocin-Induced Diabetic Rats. Transl. Res..

[295] Donmez G., Derici U., Erbas D., Arinsoy T., Onk A., Sindel S., Hasanoglu E. (2002). The Effects of Losartan and Enalapril Therapies on the Levels of Nitric Oxide, Malondialdehyde, and Glutathione in Patients with Essential Hypertension. Jpn. J. Physiol..

[296] Flammer A.J., Hermann F., Wiesli P., Schwegler B., Chenevard R., Hürlimann D., Sudano I., Gay S., Neidhart M., Riesen W. (2007). Effect of Losartan, Compared with Atenolol, on Endothelial Function and Oxidative Stress in Patients with Type 2 Diabetes and Hypertension. J. Hypertens..

[297] Cal L.A., Maso L.D., Caielli P., Pagnin E., Fusaro M., Davis P.A., Pessina A.C. (2011). Effect of Olmesartan on Oxidative Stress in Hypertensive Patients: Mechanistic Support to Clinical Trials Derived Evidence. Blood Press..

[298] Rodríguez-Lara S.Q., Trujillo-Rangel W.A., Castillo-Romero A., Totsuka-Sutto S.E., Garcia-Cobián T.A., Cardona-Muñoz E.G., Miranda-Díaz A.G., Ramírez-Lizardo E.J., García-Benavides L. (2019). Effect of Telmisartan in the Oxidative Stress Components Induced by Ischemia Reperfusion in Rats. Oxid. Med. Cell. Longev..

[299] Belardinelli R., Solenghi M., Volpe L., Purcaro A. (2007). Trimetazidine Improves Endothelial Dysfunction in Chronic Heart Failure: An Antioxidant Effect. Eur. Heart J..

[300] Bobescu E., Marceanu L.G., Dima L., Balan A., Strempel C.G., Covaciu A. (2021). Trimetazidine Therapy in Coronary Artery Disease: The Impact on Oxidative Stress, Inflammation, Endothelial Dysfunction, and Long-Term Prognosis. Am. J. Ther..

[301] Sabatine M.S., Giugliano R.P., Keech A.C., Honarpour N., Wiviott S.D., Murphy S.A., Kuder J.F., Wang H., Liu T., Wasserman S.M. (2017). Evolocumab and Clinical Outcomes in Patients with Cardiovascular Disease. N. Engl. J. Med..

[302] Lankin V.Z., Tikhaze A.K., Viigimaa M., Chazova I.E. (2018). PCSK9 Inhibitor Causes a Decrease in the Level of Oxidatively Modified Low-Density Lipoproteins in Patients with Coronary Artery Diseases. Ter. Arkh..

[303] Qi Z., Hu L., Zhang J., Yang W., Liu X., Jia D., Yao Z., Chang L., Pan G., Zhong H. (2021). PCSK9 (Proprotein Convertase Subtilisin/Kexin 9) Enhances Platelet Activation, Thrombosis, and Myocardial Infarct Expansion by Binding to Platelet CD36. Circulation.

[304] Huang G., Lu X., Zhou H., Li R., Huang Q., Xiong X., Luo Z., Li W. (2022). PCSK9 Inhibition Protects against Myocardial Ischemia-Reperfusion Injury via Suppressing Autophagy. Microvasc. Res..

[305] Młynarska E., Hajdys J., Czarnik W., Fularski P., Leszto K., Majchrowicz G., Lisińska W., Rysz J., Franczyk B. (2024). The Role of Antioxidants in the Therapy of Cardiovascular Diseases—A Literature Review. Nutrients.

[306] Jorat M.V., Tabrizi R., Mirhosseini N., Lankarani K.B., Akbari M., Heydari S.T., Mottaghi R., Asemi Z. (2018). The Effects of Coenzyme Q10 Supplementation on Lipid Profiles among Patients with Coronary Artery Disease: A Systematic Review and Meta-Analysis of Randomized Controlled Trials. Lipids Health Dis..

[307] Iqbal I., Wilairatana P., Saqib F., Nasir B., Wahid M., Latif M.F., Iqbal A., Naz R., Mubarak M.S. (2023). Plant Polyphenols and Their Potential Benefits on Cardiovascular Health: A Review. Molecules.

[308] Singh A.P., Singh R., Verma S.S., Rai V., Kaschula C.H., Maiti P., Gupta S.C. (2019). Health Benefits of Resveratrol: Evidence from Clinical Studies. Med. Res. Rev..

[309] Ciccone M.M., Cortese F., Gesualdo M., Carbonara S., Zito A., Ricci G., Pascalis F.D., Scicchitano P., Riccioni G. (2013). Dietary Intake of Carotenoids and Their Antioxidant and Anti-Inflammatory Effects in Cardiovascular Care. Mediat. Inflamm..

[310] Suzuki K., Ishii J., Kitagawa F., Kuno A., Kusuhara Y., Ochiai J., Ichino N., Osakabe K., Sugimoto K., Yamada H. (2013). Association of Serum Carotenoid Levels with N-Terminal pro-Brain-Type Natriuretic Peptide: A Cross-Sectional Study in Japan. J. Epidemiol..

[311] Abdel-Daim M.M., Zakhary N.I., Aleya L., Bungǎu S.G., Bohara R.A., Siddiqi N.J. (2018). Aging, Metabolic, and Degenerative Disorders: Biomedical Value of Antioxidants. Oxidative Med. Cell. Longev..

[312] Abdel-Daim M.M., Abo-EL-Sooud K., Aleya L., Bungǎu S.G., Najda A., Saluja R. (2018). Alleviation of Drugs and Chemicals Toxicity: Biomedical Value of Antioxidants. Oxidative Med. Cell. Longev..

